# DNA barcode *trnH*-*psbA* is a promising candidate for efficient identification of forage legumes and grasses

**DOI:** 10.1186/s13104-020-4897-5

**Published:** 2020-01-17

**Authors:** Miguel Loera-Sánchez, Bruno Studer, Roland Kölliker

**Affiliations:** Molecular Plant Breeding, Institute of Agricultural Sciences, ETH Zurich, Universitaetstrasse 2, 8092 Zurich, Switzerland

**Keywords:** DNA barcoding, Forages, Grasslands, Species richness

## Abstract

**Objective:**

Grasslands are widespread ecosystems that fulfil many functions. Plant species richness (PSR) is known to have beneficial effects on such functions and monitoring PSR is crucial for tracking the effects of land use and agricultural management on these ecosystems. Unfortunately, traditional morphology-based methods are labor-intensive and cannot be adapted for high-throughput assessments. DNA barcoding could aid increasing the throughput of PSR assessments in grasslands. In this proof-of-concept work, we aimed at determining which of three plant DNA barcodes (*rbcLa*, *matK* and *trnH*-*psbA*) best discriminates 16 key grass and legume species common in temperate sub-alpine grasslands.

**Results:**

Barcode *trnH*-*psbA* had a 100% correct assignment rate (CAR) in the five analyzed legumes, followed by *rbcLa* (93.3%) and *matK* (55.6%). Barcode *trnH*-*psbA* had a 100% CAR in the grasses *Cynosurus cristatus*, *Dactylis glomerata* and *Trisetum flavescens*. However, the closely related *Festuca, Lolium* and *Poa* species were not always correctly identified, which led to an overall CAR in grasses of 66.7%, 50.0% and 46.4% for *trnH*-*psbA*, *matK* and *rbcLa*, respectively. Barcode *trnH*-*psbA* is thus the most promising candidate for PSR assessments in permanent grasslands and could greatly support plant biodiversity monitoring on a larger scale.

## Introduction

Grasslands are some of the most widespread ecosystems on Earth, covering two-fifth of its land surface [1]. They provide roughage for ruminant livestock production and many other environmental services related to carbon sequestration, water flow regulation and soil stabilization [2, 3]. Plant species richness (PSR) is a component of biodiversity with major effects on the ecosystem functioning of grasslands. In experimental grassland plant communities, high levels of PSR stabilize yields and confer tolerance against environmental stressors [4]. Similar effects have been observed in semi-natural grasslands, which are composed of a limited number of species and are an important component of sustainable livestock production [5]. Assessing PSR is thus crucial for tracking its changes and effects on ecosystem services. However, such assessments have traditionally relied on morphology-based surveys that are labor-intensive and require trained taxonomists, limiting their use for surveying PSR over large scales and long time periods [3]. Furthermore, grasses and legumes (the two plant families of major economic relevance in temperate grasslands) can be taxonomically assessed with highest precision only when certain distinctive morphological characters are on display (e.g., flowering bodies and leaves). Still, some grass and legume species are difficult to distinguish from closely related species. A standardized, precise, high-throughput solution for PSR surveys in grasslands is therefore desirable for large-scale assessments of changes in PSR.

DNA barcoding is a methodology that has been successfully applied for standardizing and increasing the throughput of PSR surveys in ecological studies [6, 7]. DNA barcodes are organellar or nuclear loci that show a high degree of species-level conservation [8, 9]. By comparing newly sequenced DNA barcodes to reference databases, it is possible to assign an unknown biological sample to its correct taxonomy. An international effort is currently in place to maintain a well-curated, public reference database of DNA barcodes (The Barcode Of Life Datasystems database, BOLD [10]).

In animals, the DNA barcode of choice is the mitochondrial *COI* gene, which can reproducibly differentiate most of the major animal phyla [8]. In plants, in contrast, there is no single DNA barcode with comparable success [11]. Most plant DNA barcodes are located in the chloroplast genome, either within coding sequences (such as *rbcLa* and *matK*) or in intergenic regions (such as *trnH*-*psbA*) [11, 12], although some nuclear loci have also been used as DNA barcodes, e.g., the internal transcribed spacer of the ribosomal DNA (ITS) [13]. More than one barcode per plant individual are typically sequenced and used for taxonomical assignments [11, 12]. However, sequencing more than one DNA barcode per plant may not be technically feasible in higher throughput settings, particularly when analyzing mixed-species samples.

The aim of the present study was to determine the best DNA barcode sequences for forage species by screening the BOLD database for promising candidates and sequencing three DNA barcodes (*rbcLa*, *matK* and *trnH*-*psbA*) from multiple cultivars of 16 forage plant species that are common in sub-alpine grasslands.

## Main text

### Methods

#### Plant material and DNA extraction

Seeds of 2–3 cultivars of 16 forage species (*Alopecurus pratensis* L.*, Arrhenaterum elatius* L.*, Cynosurus cristatus* L.*, Dactylis glomerata* L.*, Festuca pratensis* Huds.*, F. rubra* L.*, Lolium perenne* L.*, L. multiflorum* Lam.*, Lotus corniculatus* L.*, Medicago sativa* L.*, Phleum pratense* L.*, Poa pratensis* L.*, Trifolium pratense* L.*, T. repens* L. *and Trisetum flavescens* L.), kindly provided by Agroscope, Zurich, Switzerland were used for the study (Table 1). Seeds were germinated and transferred into pot trays (77 wells, 50 cm × 32 cm, with compost as substrate). The species selected are predominant components of sub-alpine grasslands and hold great potential for multifunctional, species-rich agriculture [14, 15]. Plants were grown for 3 weeks after which DNA was extracted from three plants per species. For grasses, three leaf fragments of ~ 1 cm and for legumes three young leaflets were harvested. The plant material was freeze-dried for 48 h and pulverized in a QIAGEN TissueLyser II (QIAGEN, Hilden, Germany). DNA was extracted using the NucleoSpin^®^ II kit (Macherey–Nagel, Düren, Germany) and its integrity visually inspected by agarose gel electrophoresis (1% w/v). DNA purity and concentration were determined with a NanoDrop™ spectrophotometer (ThermoFisher Scientific, Waltham, MA, USA).

**Table 1:** Size of barcode sequences obtained for 48 plants from 16 different species of forage grasses and legumes

BOLD Process ID	Species	Cultivar	Sequence size in bp [number of n’s in sequence]		
			*rbcLa*	*matK*	*trnH*-*psbA*
SWFRG013-19	*Alopecurus pratensis*	‘Alko’ (Saatszucht Steinach, DE)	0^a^	838 [0]	0^a^
SWFRG014-19	*Alopecurus pratensis*	‘Alopex’ (Agroscope, CH)	0^a^	663 [0]	0^a^
SWFRG029-19	*Alopecurus pratensis*	‘Alopex’ (Agroscope, CH)	552 [0]	185 [0]	569 [0]
SWFRG015-19	*Arrhenatherum elatius*	‘Arone’ (Saatszucht Steinach, DE)	549 [0]	436 [0]	512 [0]
SWFRG016-19	*Arrhenatherum elatius*	‘Median’ (DLF Životice, CZ)	550 [0]	610 [0]	0^a^
SWFRG031-19	*Arrhenatherum elatius*	‘Median’ (DLF Životice, CZ)	0^a^	825 [0]	0^a^
SWFRG030-19	*Cynosurus cristatus*	‘Cresta’ (Agroscope, CH)	541 [0]	513 [0]	466 [0]
SWFRG045-19	*Cynosurus cristatus*	‘Lena’ (HBLF, AT)	585 [0]	531 [0]	564 [0]
SWFRG046-19	*Cynosurus cristatus*	‘Rožnovská’ (OSEVA PRO, CZ)	529 [0]	870 [0]	569 [0]
SWFRG001-19	*Dactylis glomerata*	‘Barexcel’ (Barenbrug, NL)	577 [0]	640 [0]	519 [0]
SWFRG002-19	*Dactylis glomerata*	‘Brennus’ (R2n, FR)	546 [0]	865 [13]	576 [0]
SWFRG017-19	*Dactylis glomerata*	‘Reda’ (Agroscope, CH)	534 [0]	866 [13]	561 [0]
SWFRG003-19	*Festuca pratensis*	‘Cosmolit’ (Saatszucht Steinach, DE)	547 [0]	579 [0]	558 [0]
SWFRG004-19	*Festuca pratensis*	‘Paradisia’ (Agroscope, CH)	549 [0]	888 [7]	566 [0]
SWFRG019-19	*Festuca pratensis*	‘Pradel’ (Agroscope, CH)	552 [0]	590 [0]	553 [0]
SWFRG007-19	*Festuca rubra*	‘Echo’ (DLF-Trifolium, DK)	559 [0]	886 [3]	274 [21]
SWFRG008-19	*Festuca rubra*	‘Pran Solas’ (Schweizer, CH)	588 [0]	869 [0]	570 [0]
SWFRG023-19	*Festuca rubra*	‘Roland’ (Saatszucht Steinach, DE)	558 [0]	543 [0]	594 [0]
SWFRG005-19	*Lolium multiflorum*	‘Axis’ (Agroscope, CH)	581 [0]	874 [0]	551 [0]
SWFRG006-19	*Lolium multiflorum*	‘Caribu’ (Agroscope, CH)	577 [0]	884 [8]	567 [7]
SWFRG021-19	*Lolium multiflorum*	‘Zebra’ (Agroscope, CH)	571 [0]	586 [0]	551 [0]
SWFRG009-19	*Lolium perenne*	‘Arara’ (Agroscope, CH)	547 [0]	883 [3]	539 [5]
SWFRG010-19	*Lolium perenne*	‘Arvella’ (Agroscope, CH)	582 [0]	481 [0]	567 [15]
SWFRG025-19	*Lolium perenne*	‘Lipresso’ (Euro Grass, DE)	488 [0]	835 [0]	614 [0]
SWFRG024-19	*Lotus corniculatus*	‘Lotar’ (OSEVA UNI, SK)	544 [0]	399 [0]	294 [0]
SWFRG039-19	*Lotus corniculatus*	‘Lotar’ (OSEVA UNI, SK)	548 [0]	509 [0]	414 [0]
SWFRG040-19	*Lotus corniculatus*	‘Polom’ (CVRV, VÚRV, CZ)	502 [0]	702 [0]	412 [0]
SWFRG022-19	*Medicago sativa*	‘Artemis’ (Barenbrug, NL)	580 [0]	410 [0]	268 [14]
SWFRG037-19	*Medicago sativa*	‘Catera’ (Saatszucht Steinach, DE)	526 [0]	435 [0]	438 [3]
SWFRG038-19	*Medicago sativa*	‘Sanditi’ (Barenbrug, NL)	548 [0]	432 [0]	445 [2]
SWFRG028-19	*Onobrychis viciifolia*	‘Perdix’ (Agroscope, CH)	550 [0]	576 [0]	289 [0]
SWFRG043-19	*Onobrychis viciifolia*	‘Perly’ (Agroscope, CH)	582 [0]	627 [0]	284 [0]
SWFRG044-19	*Onobrychis viciifolia*	‘Višňovský’ (Agrogen, CZ)	543 [0]	694 [10]	287 [0]
SWFRG032-19	*Phleum pratense*	‘Anjo’ (ILVO, BE)	540 [0]	0^a^	0^a^
SWFRG047-19	*Phleum pratense*	‘Tiller’ (DLF-Trifolium, DK)	576 [0]	527 [5]	584 [0]
SWFRG048-19	*Phleum pratense*	‘Toro’ (CRA-FLC, IT)	0^a^	516 [1]	0^a^
SWFRG011-19	*Poa pratensis*	‘Likollo’ (DSV, DE)	470 [0]	865 [0]	540 [0]
SWFRG012-19	*Poa pratensis*	‘Nixe’ (Saatszucht Steinach, DE)	571 [0]	868 [0]	576 [0]
SWFRG027-19	*Poa pratensis*	‘Tommy’ (DLF-Trifolium, DK)	0^a^	489 [0]	0^a^
SWFRG020-19	*Trifolium pratense*	‘Bonus’ (Selgen, CZ)	549 [0]	0^a^	410 [0]
SWFRG035-19	*Trifolium pratense*	‘Diplomat’ (DSV, DE)	564 [0]	556 [0]	485 [0]
SWFRG036-19	*Trifolium pratense*	‘Pavo’ (Agroscope, CH)	514 [0]	430 [0]	496 [0]
SWFRG026-19	*Trifolium repens*	‘Beaumont’ (CW 090; Barenbrug, NL)	550 [0]	419 [0]	448 [0]
SWFRG041-19	*Trifolium repens*	‘Bombus’ (Agroscope, CH)	579 [0]	481 [0]	471 [0]
SWFRG042-19	*Trifolium repens*	‘Hebe’ (Svalöf-Weibull, SE)	571 [0]	444 [0]	447 [0]
SWFRG018-19	*Trisetum flavescens*	‘Gunther’ (HBLFA, AT)	504 [0]	859 [6]	570 [0]
SWFRG033-19	*Trisetum flavescens*	‘Gunther’ (HBLFA, AT)	575 [0]	586 [4]	571 [0]
SWFRG034-19	*Trisetum flavescens*	‘Trisett51’ (Saatszucht Steinach, DE)	558 [0]	887 [4]	568 [0]
	Total sequences		43 (89.58%)	46 (95.83%)	41 (85.42%)

^**a**^ Repeatedly unsuccessful PCR

#### DNA barcode amplification and sequencing

The BOLD database was screened for DNA barcode sequences of the selected species and close relatives; barcodes *rbcLa*, *matK* and *trnH*-*psbA* were selected as candidates because they reported the most available sequences. Those DNA barcodes are mainly located in the chloroplast genome and are not known to have paralogs that can interfere with taxonomic assignments, as is the case for some nuclear loci such as ITS [13]. Primer sequences for the three barcodes were obtained from BOLD [10] and were optimized for amplification in the target plant families (Additional file 1: Table S1). Each PCR reaction consisted of 15 ng of template DNA, 1× flexi buffer (Promega, Madison, WI, USA), 2 mM MgCl_2_, 200 µM dNTPs, each primer at 0.4 µM, 0.75 units of GoTaq^®^ G2 Flexi DNA Polymerase (Promega, Madison, WI, USA) and water to a final volume of 30 µL.

For *rbcLa,* PCR conditions were 5 min at 94 °C followed by 33 cycles of 40 s at 94 °C, 1 min at 55 °C and 40 s at 72 °C, followed by a final extension cycle of 10 min at 72 °C. For *matK* and *trnH*-*psbA*, a 5 min at 94 °C followed by 50 cycles of 40 s at 94 °C, 1 min at 54 °C and 40 s at 72 °C followed by a final extension cycle of 10 min at 72 °C were used. The integrity of the amplicons was visually inspected by agarose gel electrophoresis (1% w/v).

Amplicons were purified in a MultiScreen PCR96 filter plate (Merck, Darmstadt, Germany). Sequencing reactions were prepared with 1× BigDye™ Terminator 3.1 Reaction Mix (ThermoFisher Scientific, Waltham, MA, USA), 1× BigDye™ 3.1 Sequencing Buffer, forward or reverse primer at 0.16 µM and 800 ng of purified amplicon to a final volume of 5 µL. The same primers used for PCR were used for sequencing. Capillary electrophoresis was performed on a 3130 ABI (ThermoFisher Scientific, Waltham, MA, USA). The resulting traces were quality filtered and merged using GAP4 [16] with the default settings. All traces and sequences were uploaded to BOLD v4 (project code: SWFRG; http://www.boldsystems.org/index.php/Public_SearchTerms).

#### Taxonomical assignments

Sequences of *matK*, *rbcLa* and *trnH*-*psbA* were downloaded from BOLD v4 on May 23, 2019 [10]. Only sequences from the Poaceae and Fabaceae families with no contaminants and longer than 200 bp were included. In total, 6232 *rbcLa,* 11,971 *matK* and 1236 *trnH*-*psbA* sequences were present in the downloaded fasta files, which also include the plants from the BOLD project SWFRG (Additional file 1: Table S2). The taxonomical identifiers of the BOLD fasta files were reformatted to remove spaces and rearrange their informative fields in a consistent manner (*fasta_name_reformat.py* script from https://github.com/mloera/forage-barcoding).

Each barcode-specific fasta file was then used to make a blast database and the SWFRG sequences were queried in their corresponding database with blastn using the flag *outfmt* = 6 (i.e., tabular format). The resulting blast output tables were parsed with the *blastn_matcher.R* script from the above-mentioned GitHub repository. The script removes self-hits and corrects some misspellings in the taxonomy of queries and hits. The script then compares the taxonomy of the queries and hits at the species- and genus-level. A “match” was called when the taxonomy of a query sequence is equal to the taxonomy of the highest scoring hit or hits (Additional file 1: Table S3). A “taxonomical assignment rate” for each barcode was then calculated as the ratio between the sum of its correct taxonomical assignments and the total number of query sequences.

### Results and discussion

#### PCR and sequencing results

The primer sequences of *trnH*-*psbA* and *matK* were adapted to allow for amplification within the target species, while the primer sequences of *rbcLa* did not need any modification (Additional file 1: Table S1). From the 48 processed specimens, 130 sequences were obtained (46 for *matK*, 43 for *rbcLa* and 41 for *trnH*-*psbA*-) after repeating and optimizing failed amplifications. The size of the sequences ranged from 470 to 588 bp for *rbcLa*, 185 to 888 bp for *matK* and 268 to 614 bp for *trnH*-*psbA* (Table 1).

#### Taxonomical assignments

Barcode *trnH*-*psbA* had a 100% correct assignment rate (CAR) in legumes, followed by *rbcLa* (93.3%) *matK* (57.1%; Table 2). The highest CAR for grasses was 65.4% with *trnH*-*psbA*, followed by *matK* (48.4%) and *rbcLa* (46.4%). Overall, genus-level CARs were 69.8%, 73.3% and 90.2% for *rbcLa*, *matK* and *trnH*-*psbA*, respectively. Legumes had also the highest assignment rate on the genus level (100% correct assignments for all barcodes; Table 2), while correct assignments for grass genera were 53.6%, 61.3% and 84.6% for barcodes *rbcLa*, *matK* and *trnH*-*psbA*, respectively.

**Table 2:** Species- and genus-level assignment success by barcode

Barcode	Species-level assignment rate			Genus-level assignment rate		
	Overall (%)	Grasses (%)	Legumes (%)	Overall (%)	Grasses (%)	Legumes (%)
*rbcLa*	62.8	46.4	93.3	69.8	53.6	100.0
*matK*	51.1	48.4	57.1	73.3	61.3	100.00
*trnH*-*psbA*	78.0	65.4	100.0	90.2	84.6	100.0

The low CARs for grass DNA barcodes could be due to various factors. Some grass species, such as *Poa* spp., are notoriously hard to discriminate morphologically and their phylogeny is subject to controversy [17, 18]. This could have resulted in misidentified reference sequences. Another factor is the high genetic similarity between some grass taxa. For example, the genetic similarity of some species of the *Festuca*-*Lolium* complex is reported to be > 90%, as calculated from transcriptomic data of orthologous genes [19]. This may result in a higher proportion of incorrect taxonomic assignments for such grass species [20].

Barcode *trnH*-*psbA* makes for a good candidate for large-scale DNA barcoding of forage legumes and some grasses, such as *C. cristatus*, *D. glomerata* and *T. flavescens* (Table 3). However, further work is needed to produce reference sequences in more forage species and cultivars. Overall, our results provide the basic tools to implement DNA barcoding in forage species (i.e., family-specific primer pairs and a standard bioinformatic workflow for taxonomic assignments) and can help in choosing an appropriate DNA barcode for high-throughput applications. Such high-throughput applications could greatly enhance the biodiversity-monitoring protocols that are used to study the ecology of grasslands, its dynamics and its interplay with agriculture.

**Table 3:** Species-level taxonomic assignment success by family, query species and barcode sequence

Family	Query species	*matK*	*rbcLa*	*trnH*-*psbA*
Poaceae	*Alopecurus pratensis*	0/3	0/1	0/1
	*Arrhenatherum elatius*	1/3	0/2	1/1
	*Cynosurus cristatus*	*3/3*	2/3	*3/3*
	*Dactylis glomerata*	*3/3*	2/3	*3/3*
	*Festuca pratensis*	2/3	0/3	1/3
	*Festuca rubra*	1/3	*3/3*	2/3
	*Lolium multiflorum*	0/3	2/3	1/3
	*Lolium perenne*	2/3	0/3	2/3
	*Phleum pratense*	1/2	1/2	1/1
	*Poa pratensis*	0/3	1/2	0/2
	*Trisetum flavescens*	2/3	2/3	*3/3*
Fabaceae	*Lotus corniculatus*	1/3	*3/3*	*3/3*
	*Medicago sativa*	2/3	*3/3*	*3/3*
	*Onobrychis viciifolia*	2/3	*3/3*	*3/3*
	*Trifolium pratense*	2/2	2/3	*3/3*
	*Trifolium repens*	1/3	*3/3*	*3/3*

Italics indicate 100% taxonomic assignment success

## Limitations

This is exploratory work focused on the most common forage plant species from sub-alpine temperate grasslands; further work is needed to address other forage species from different kinds of grasslands.

As a proof of concept, three specimens per species were analyzed.

## Supplementary information

**Additional file 1**: **Table S1.** PCR primers used in this study. **Table S2.** Overview of the Barcoding of Life Datasystems (BOLD, [10]) reference barcode sequences used for taxonomical assignments. **Table S3.** Highest scoring blastn hits for the plant specimens of the BOLD project “SWFRG”.

## Data Availability

The datasets generated and/or analysed during the current study are available in the following GitHub repository: https://github.com/mloera/forage-barcoding. Sequencing trace files are found in BOLD (http://www.boldsystems.org/index.php/Public_SearchTerms) using the search term “SWFRG” and are also available on 10.5281/zenodo.3597069.

## Abbreviations

BOLD: Barcode of Life Datasystems
CAR: correct assignment rate
PSR: plant species richness

## References

[1] Reynolds SG. Chapter 1 Introduction. In: Suttie JM, Reynolds SG, Batello C, editors. Grasslands of the World. Food and Agriculture Organization of the United Nations; 2005. http://www.fao.org/3/y8344e00.htm.

[2] Lindborg R, Bengtsson J, Berg Å, Cousins SAO, Eriksson O, Gustafsson T (2008). A landscape perspective on conservation of semi-natural grasslands. Agric Ecosyst Environ.

[3] Bengtsson J, Bullock JM, Egoh B, Everson C, Everson T, O’Connor T (2019). Grasslands-more important for ecosystem services than you might think. Ecosphere..

[4] Wang Y, Cadotte MW, Chen Y, Fraser LH, Zhang Y, Huang F (2019). Global evidence of positive biodiversity effects on spatial ecosystem stability in natural grasslands. Nat Commun..

[5] Feßel C, Meier IC, Leuschner C (2016). Relationship between species diversity, biomass and light transmittance in temperate semi-natural grasslands: is productivity enhanced by complementary light capture?. J Veg Sci.

[6] Kress WJ (2017). Plant DNA barcodes: applications today and in the future. J Syst Evol..

[7] Kress WJ, García-Robledo C, Uriarte M, Erickson DL (2015). DNA barcodes for ecology, evolution, and conservation. Trends Ecol Evol.

[8] Hebert PDN, Ratnasingham S, de Waard JR (2003). Barcoding animal life: cytochrome c oxidase subunit 1 divergences among closely related species. Proc R Soc London Ser B Biol Sci..

[9] Hebert PDN, Cywinska A, Ball SL, DeWaard JR (2003). Biological identifications through DNA barcodes. Proc R Soc London Ser B Biol Sci..

[10] RATNASINGHAM SUJEEVAN, HEBERT PAUL D. N. (2007). BARCODING: bold: The Barcode of Life Data System (http://www.barcodinglife.org). Molecular Ecology Notes.

[11] CBOL Plant Working Group (2009). A DNA barcode for land plants. Proc Natl Acad Sci USA..

[12] Kress WJ, Erickson DL (2007). A two-locus global DNA barcode for land plants: the coding *rbcL* gene complements the non-coding *trnH*-*psbA* spacer region. PLoS ONE.

[13] Bolson M, Smidt E, de C, Brotto ML, Silva-Pereira V (2015). ITS and trnH-psbA as efficient DNA Barcodes to identify threatened commercial woody angiosperms from southern Brazilian Atlantic rainforests. PLOS ONE.

[14] French KE (2017). Species composition determines forage quality and medicinal value of high diversity grasslands in lowland England. Agric Ecosyst Environ.

[15] Lüscher A, Mueller-Harvey I, Soussana JF, Rees RM, Peyraud JL (2014). Potential of legume-based grassland-livestock systems in Europe: a review. Grass Forage Sci.

[16] Staden R, Judge DP, Bonfield JK, Krawetz SA, Womble DD (2003). Managing sequencing projects in the GAP4 environment. Introduction to bioinformatics: a theoretical and practical approach.

[17] Nosov NN, Punina EO, Machs EM, Rodionov AV (2015). Interspecies hybridization in the origin of plant species: cases in the genus *Poa* sensu lato. Biol Bull Rev..

[18] Patterson JT, Larson SR, Johnson PG (2005). Genome relationships in polyploid *Poa pratensis* and other *Poa* species inferred from phylogenetic analysis of nuclear and chloroplast DNA sequences. Genome..

[19] Czaban A, Sharma S, Byrne SL, Spannagl M, Mayer KFX, Asp T (2015). Comparative transcriptome analysis within the Lolium/Festuca species complex reveals high sequence conservation. BMC Genomics..

[20] Meyer CP, Paulay G (2005). DNA barcoding: error rates based on comprehensive sampling. PLoS Biol.

