# Supplementary material for: DNA barcode trnH-psbA is a promising candidate for efficient identification of forage legumes and grasses
Source: BMC Res Notes. 2020 Jan 17;13:35. doi: 10.1186/s13104-020-4897-5 (PMC6969398; doi:10.1186/s13104-020-4897-5)
Supplement: Supplementary file 1 — Additional file 1: Table S1. PCR primers used in this study. Table S2. Overview of the Barcoding of Life Datasystems (BOLD, [10]) reference barcode sequences used for taxonomical assignments. Table S3. Highest scoring blastn hits for the plant specimens of the BOLD project “SWFRG”. [file 13104_2020_4897_MOESM1_ESM.docx]

***Additional file 1***

***Table S1. PCR primers used in this study****.*

| **Barcode** | **Primer sequence (5’ to 3’; modifications to the original sequences are in bold and underlined)** | **Primer name (BOLD nomenclature)** | **References** |
| --- | --- | --- | --- |
|  |  |  |  |
| ***rbcLa*** | ATG TCA CCA CAA ACA GAG ACT AAA GC | rbcLa-F | [1–3] |
|  | GTA AAA TCA AGT CCA CCR CG | rbcLa-R | [4, 5] |
| ***matK*** | **G**AA TTT ACG **C**TC **T**AT TCA KTC | matK-F_Poalesv2 (modified from XF1) | [6] |
|  | **G**CT TTT ATG TTT AC**G** **A**GC **Y**AA | matK-R_Poalesv2 (modified from MatK2F-r) | [7] |
|  |  |  |  |
|  | TTC AAA CCC TTC GTT ACT GGA TVA A | FAB_F | [6] |
|  | CTT TTG TGT TTA CGA GCC AAD G | FAB_R | [6] |
| ***trnH-psbA*** | GTT ATG CAT GAA CGT AAY GCT C | psbA3_fv2 (modified from psbA3_f) | [8] |
|  | GCR TGG TGG ATT CAC AAT CC | trnHf_05v2 (modified from trnHf_05) | [9] |

***Table S2. Overview of the Barcoding of Life Datasystems (BOLD, [10]) reference barcode sequences used for taxonomical assignments***

| **Barcode** | **Genera** | **Fully identified species** | **Unidentified species** | **Min. seqs. per spp.** | **Max. seqs. per spp.** | **Total sequences** | **Minimum length in bp** |
| --- | --- | --- | --- | --- | --- | --- | --- |
|  |  |  |  |  |  |  |  |
| *rbcLa* | 822 | 2683 | 121 | 1 | 95 | 6064 | 300 |
|  |  |  |  |  |  |  |  |
| *matK* | 1086 | 4707 | 95 | 1 | 99 | 10882 | 450 |
|  |  |  |  |  |  |  |  |
| *trnH-psbA* | 151 | 359 | 8 | 1 | 32 | 1012 | 200 |
|  |  |  |  |  |  |  |  |

***Table S3. Highest scoring blastn hits for the plant specimens of the BOLD project “SWFRG”***

| QUERY.SEQID | QUERY.FAMILY | QUERY.SPECIES | REF.SEQID | REF.FAMILY | REF.SPECIES | PID | SCORE | MATCH | BARCODE |
| --- | --- | --- | --- | --- | --- | --- | --- | --- | --- |
| SWFRG001-19 | Poaceae | *Dactylis glomerata* | SWFRG045-19 | Poaceae | *Cynosurus cristatus* | 98.44 | 1016 | FALSE | rbcLa |
| SWFRG002-19 | Poaceae | *Dactylis glomerata* | SWFRG017-19 | Poaceae | *Dactylis glomerata* | 100 | 987 | TRUE | rbcLa |
| SWFRG003-19 | Poaceae | *Festuca pratensis* | SWFRG010-19 | Poaceae | *Lolium perenne* | 100 | 1003 | FALSE | rbcLa |
| SWFRG004-19 | Poaceae | *Festuca pratensis* | SWFRG009-19 | Poaceae | *Lolium perenne* | 99.63 | 996 | FALSE | rbcLa |
| SWFRG005-19 | Poaceae | *Lolium multiflorum* | SWFRG010-19 | Poaceae | *Lolium perenne* | 99.65 | 1038 | FALSE | rbcLa |
| SWFRG006-19 | Poaceae | *Lolium multiflorum* | SWFRG021-19 | Poaceae | *Lolium multiflorum* | 99.65 | 1042 | TRUE | rbcLa |
| SWFRG007-19 | Poaceae | *Festuca rubra* | VEMSH159-13 | Poaceae | *Festuca rubra* | 99.63 | 998 | TRUE | rbcLa |
| SWFRG008-19 | Poaceae | *Festuca rubra* | GRII046-08 | Poaceae | *Festuca rubra* | 99.46 | 1005 | TRUE | rbcLa |
| SWFRG008-19 | Poaceae | *Festuca rubra* | GRASS1331-08 | Poaceae | *Festuca rubra* | 99.46 | 1005 | TRUE | rbcLa |
| SWFRG009-19 | Poaceae | *Lolium perenne* | SWFRG004-19 | Poaceae | *Festuca pratensis* | 99.63 | 996 | FALSE | rbcLa |
| SWFRG010-19 | Poaceae | *Lolium perenne* | SWFRG005-19 | Poaceae | *Lolium multiflorum* | 99.65 | 1038 | FALSE | rbcLa |
| SWFRG010-19 | Poaceae | *Lolium perenne* | KSR314-07 | Poaceae | *Festuca arundinacea* | 99.82 | 1038 | FALSE | rbcLa |
| SWFRG010-19 | Poaceae | *Lolium perenne* | KSR077-07 | Poaceae | *Lolium arundinaceum* | 99.82 | 1038 | FALSE | rbcLa |
| SWFRG011-19 | Poaceae | *Poa pratensis* | SWFRG012-19 | Poaceae | *Poa pratensis* | 100 | 848 | FALSE | rbcLa |
| SWFRG011-19 | Poaceae | *Poa pratensis* | BBYUK2827-18 | Poaceae | *Poa arctica* | 100 | 848 | FALSE | rbcLa |
| SWFRG011-19 | Poaceae | *Poa pratensis* | BBYUK2821-18 | Poaceae | *Poa arctica* | 100 | 848 | FALSE | rbcLa |
| SWFRG011-19 | Poaceae | *Poa pratensis* | SDH3003-15 | Poaceae | *Poa bigelovii* | 100 | 848 | FALSE | rbcLa |
| SWFRG011-19 | Poaceae | *Poa pratensis* | SDH2967-15 | Poaceae | *Poa howellii* | 100 | 848 | FALSE | rbcLa |
| SWFRG011-19 | Poaceae | *Poa pratensis* | SDH2956-15 | Poaceae | *Poa pratensis* | 100 | 848 | FALSE | rbcLa |
| SWFRG011-19 | Poaceae | *Poa pratensis* | PCUBC840-14 | Poaceae | *Poa chaixii* | 100 | 848 | FALSE | rbcLa |
| SWFRG011-19 | Poaceae | *Poa pratensis* | PCUBC235-14 | Poaceae | *Poa laxiflora* | 100 | 848 | FALSE | rbcLa |
| SWFRG011-19 | Poaceae | *Poa pratensis* | PCUBC223-14 | Poaceae | *Poa laxiflora* | 100 | 848 | FALSE | rbcLa |
| SWFRG011-19 | Poaceae | *Poa pratensis* | SDH2500-14 | Poaceae | *Poa palustris* | 100 | 848 | FALSE | rbcLa |
| SWFRG011-19 | Poaceae | *Poa pratensis* | SDH2494-14 | Poaceae | *Poa atropurpurea* | 100 | 848 | FALSE | rbcLa |
| SWFRG011-19 | Poaceae | *Poa pratensis* | SCBI402-14 | Poaceae | *Poaceae* | 100 | 848 | FALSE | rbcLa |
| SWFRG011-19 | Poaceae | *Poa pratensis* | FBPL2879-13 | Poaceae | *Poa chaixii* | 100 | 848 | FALSE | rbcLa |
| SWFRG011-19 | Poaceae | *Poa pratensis* | FBPL2852-13 | Poaceae | *Poa chaixii* | 100 | 848 | FALSE | rbcLa |
| SWFRG011-19 | Poaceae | *Poa pratensis* | FBPL2763-13 | Poaceae | *Cinna latifolia* | 100 | 848 | FALSE | rbcLa |
| SWFRG011-19 | Poaceae | *Poa pratensis* | FBPL2630-13 | Poaceae | *Poa arctica* | 100 | 848 | FALSE | rbcLa |
| SWFRG011-19 | Poaceae | *Poa pratensis* | FBPL2629-13 | Poaceae | *Poa arctica* | 100 | 848 | FALSE | rbcLa |
| SWFRG011-19 | Poaceae | *Poa pratensis* | FBPL2394-13 | Poaceae | *Poa alpigena* | 100 | 848 | FALSE | rbcLa |
| SWFRG011-19 | Poaceae | *Poa pratensis* | FBPL2387-13 | Poaceae | *Cinna latifolia* | 100 | 848 | FALSE | rbcLa |
| SWFRG011-19 | Poaceae | *Poa pratensis* | FBPL2350-13 | Poaceae | *Alopecurus geniculatus* | 100 | 848 | FALSE | rbcLa |
| SWFRG011-19 | Poaceae | *Poa pratensis* | FBPL2335-13 | Poaceae | *Poa alpigena* | 100 | 848 | FALSE | rbcLa |
| SWFRG011-19 | Poaceae | *Poa pratensis* | FBPL2292-13 | Poaceae | *Poa angustifolia* | 100 | 848 | FALSE | rbcLa |
| SWFRG011-19 | Poaceae | *Poa pratensis* | FBPL2271-13 | Poaceae | *Poa subcaerulea* | 100 | 848 | FALSE | rbcLa |
| SWFRG011-19 | Poaceae | *Poa pratensis* | FBPL2267-13 | Poaceae | *Alopecurus geniculatus* | 100 | 848 | FALSE | rbcLa |
| SWFRG011-19 | Poaceae | *Poa pratensis* | FBPL2216-13 | Poaceae | *Poa alpigena* | 100 | 848 | FALSE | rbcLa |
| SWFRG011-19 | Poaceae | *Poa pratensis* | FBPL2187-13 | Poaceae | *Alopecurus geniculatus* | 100 | 848 | FALSE | rbcLa |
| SWFRG011-19 | Poaceae | *Poa pratensis* | FBPL2177-13 | Poaceae | *Poa pratensis* | 100 | 848 | FALSE | rbcLa |
| SWFRG011-19 | Poaceae | *Poa pratensis* | FBPL2161-13 | Poaceae | *Poa subcaerulea* | 100 | 848 | FALSE | rbcLa |
| SWFRG011-19 | Poaceae | *Poa pratensis* | FBPL2120-13 | Poaceae | *Poa angustifolia* | 100 | 848 | FALSE | rbcLa |
| SWFRG011-19 | Poaceae | *Poa pratensis* | FBPL2061-13 | Poaceae | *Poa arctica* | 100 | 848 | FALSE | rbcLa |
| SWFRG011-19 | Poaceae | *Poa pratensis* | FBPL2048-13 | Poaceae | *Cinna latifolia* | 100 | 848 | FALSE | rbcLa |
| SWFRG011-19 | Poaceae | *Poa pratensis* | FBPL1989-13 | Poaceae | *Poa remota* | 100 | 848 | FALSE | rbcLa |
| SWFRG011-19 | Poaceae | *Poa pratensis* | FBPL1988-13 | Poaceae | *Poa pratensis* | 100 | 848 | FALSE | rbcLa |
| SWFRG011-19 | Poaceae | *Poa pratensis* | FBPL1945-13 | Poaceae | *Poa chaixii* | 100 | 848 | FALSE | rbcLa |
| SWFRG011-19 | Poaceae | *Poa pratensis* | FBPL1856-13 | Poaceae | *Poa pratensis* | 100 | 848 | FALSE | rbcLa |
| SWFRG011-19 | Poaceae | *Poa pratensis* | FBPL1855-13 | Poaceae | *Poa subcaerulea* | 100 | 848 | FALSE | rbcLa |
| SWFRG011-19 | Poaceae | *Poa pratensis* | FBPL1854-13 | Poaceae | *Poa remota* | 100 | 848 | FALSE | rbcLa |
| SWFRG011-19 | Poaceae | *Poa pratensis* | FBPL1519-13 | Poaceae | *Poa remota* | 100 | 848 | FALSE | rbcLa |
| SWFRG011-19 | Poaceae | *Poa pratensis* | FBPL1449-13 | Poaceae | *Alopecurus aequalis* | 100 | 848 | FALSE | rbcLa |
| SWFRG011-19 | Poaceae | *Poa pratensis* | FBPL1295-13 | Poaceae | *Alopecurus aequalis* | 100 | 848 | FALSE | rbcLa |
| SWFRG011-19 | Poaceae | *Poa pratensis* | WABLK287-13 | Poaceae | *Cinna latifolia* | 100 | 848 | FALSE | rbcLa |
| SWFRG011-19 | Poaceae | *Poa pratensis* | WABLK276-13 | Poaceae | *Cinna latifolia* | 100 | 848 | FALSE | rbcLa |
| SWFRG011-19 | Poaceae | *Poa pratensis* | VPSBC476-13 | Poaceae | *Poa marcida* | 100 | 848 | FALSE | rbcLa |
| SWFRG011-19 | Poaceae | *Poa pratensis* | GRAFW2133-13 | Poaceae | *Poa hartzii* | 100 | 848 | FALSE | rbcLa |
| SWFRG011-19 | Poaceae | *Poa pratensis* | GRAFW2069-13 | Poaceae | *Alopecurus borealis* | 100 | 848 | FALSE | rbcLa |
| SWFRG011-19 | Poaceae | *Poa pratensis* | GRAFW2057-13 | Poaceae | *Poa arctica* | 100 | 848 | FALSE | rbcLa |
| SWFRG011-19 | Poaceae | *Poa pratensis* | GRAFW2007-13 | Poaceae | *Poa pratensis* | 100 | 848 | FALSE | rbcLa |
| SWFRG011-19 | Poaceae | *Poa pratensis* | GRAFW2004-13 | Poaceae | *Poa arctica* | 100 | 848 | FALSE | rbcLa |
| SWFRG011-19 | Poaceae | *Poa pratensis* | GRAFW1907-13 | Poaceae | *Alopecurus borealis* | 100 | 848 | FALSE | rbcLa |
| SWFRG011-19 | Poaceae | *Poa pratensis* | VEMSH158-13 | Poaceae | *Poa pratensis* | 100 | 848 | FALSE | rbcLa |
| SWFRG011-19 | Poaceae | *Poa pratensis* | VEMSH091-13 | Poaceae | *Poa pratensis* | 100 | 848 | FALSE | rbcLa |
| SWFRG011-19 | Poaceae | *Poa pratensis* | REDNO049-12 | Poaceae | *Cinna latifolia* | 100 | 848 | FALSE | rbcLa |
| SWFRG011-19 | Poaceae | *Poa pratensis* | HIMS1861-12 | Poaceae | *Poa fendleriana* | 100 | 848 | FALSE | rbcLa |
| SWFRG011-19 | Poaceae | *Poa pratensis* | HIMS1645-12 | Poaceae | *Poa nervosa* | 100 | 848 | FALSE | rbcLa |
| SWFRG011-19 | Poaceae | *Poa pratensis* | MKTRT143-12 | Poaceae | *Vulpia octoflora* | 100 | 848 | FALSE | rbcLa |
| SWFRG011-19 | Poaceae | *Poa pratensis* | MKTRT142-12 | Poaceae | *Vulpia octoflora* | 100 | 848 | FALSE | rbcLa |
| SWFRG011-19 | Poaceae | *Poa pratensis* | MSTH046-12 | Poaceae | *Poa saltuensis* | 100 | 848 | FALSE | rbcLa |
| SWFRG011-19 | Poaceae | *Poa pratensis* | WAT360-12 | Poaceae | *Poa pratensis* | 100 | 848 | FALSE | rbcLa |
| SWFRG011-19 | Poaceae | *Poa pratensis* | BBYUK2092-12 | Poaceae | *Poa macrantha* | 100 | 848 | FALSE | rbcLa |
| SWFRG011-19 | Poaceae | *Poa pratensis* | BBYUK2082-12 | Poaceae | *Poa arctica* | 100 | 848 | FALSE | rbcLa |
| SWFRG011-19 | Poaceae | *Poa pratensis* | BBYUK2081-12 | Poaceae | *Poa porsildii* | 100 | 848 | FALSE | rbcLa |
| SWFRG011-19 | Poaceae | *Poa pratensis* | BBYUK2080-12 | Poaceae | *Poa porsildii* | 100 | 848 | FALSE | rbcLa |
| SWFRG011-19 | Poaceae | *Poa pratensis* | BBYUK2078-12 | Poaceae | *Poa cusickii* | 100 | 848 | FALSE | rbcLa |
| SWFRG011-19 | Poaceae | *Poa pratensis* | PLPAR012-11 | Poaceae | *Cinna latifolia* | 100 | 848 | FALSE | rbcLa |
| SWFRG011-19 | Poaceae | *Poa pratensis* | PLKEN290-11 | Poaceae | *Poa pratensis* | 100 | 848 | FALSE | rbcLa |
| SWFRG011-19 | Poaceae | *Poa pratensis* | PLCOC134-11 | Poaceae | *Cinna latifolia* | 100 | 848 | FALSE | rbcLa |
| SWFRG011-19 | Poaceae | *Poa pratensis* | PLTHU150-11 | Poaceae | *Poa pratensis* | 100 | 848 | FALSE | rbcLa |
| SWFRG011-19 | Poaceae | *Poa pratensis* | FCA2986-11 | Poaceae | *Poa paucispicula* | 100 | 848 | FALSE | rbcLa |
| SWFRG011-19 | Poaceae | *Poa pratensis* | FCA2985-11 | Poaceae | *Poa paucispicula* | 100 | 848 | FALSE | rbcLa |
| SWFRG011-19 | Poaceae | *Poa pratensis* | FCA2984-11 | Poaceae | *Poa paucispicula* | 100 | 848 | FALSE | rbcLa |
| SWFRG011-19 | Poaceae | *Poa pratensis* | FCA2844-11 | Poaceae | *Alopecurus borealis* | 100 | 848 | FALSE | rbcLa |
| SWFRG011-19 | Poaceae | *Poa pratensis* | FCA2824-11 | Poaceae | *Poa arctica* | 100 | 848 | FALSE | rbcLa |
| SWFRG011-19 | Poaceae | *Poa pratensis* | FCA2520-11 | Poaceae | *Poa arctica* | 100 | 848 | FALSE | rbcLa |
| SWFRG011-19 | Poaceae | *Poa pratensis* | FCA2490-11 | Poaceae | *Poa arctica* | 100 | 848 | FALSE | rbcLa |
| SWFRG011-19 | Poaceae | *Poa pratensis* | FCA2488-11 | Poaceae | *Poa pratensis* | 100 | 848 | FALSE | rbcLa |
| SWFRG011-19 | Poaceae | *Poa pratensis* | FCA2359-11 | Poaceae | *Poa pratensis* | 100 | 848 | FALSE | rbcLa |
| SWFRG011-19 | Poaceae | *Poa pratensis* | FCA2223-11 | Poaceae | *Poa palustris* | 100 | 848 | FALSE | rbcLa |
| SWFRG011-19 | Poaceae | *Poa pratensis* | FCA2155-11 | Poaceae | *Poa arctica* | 100 | 848 | FALSE | rbcLa |
| SWFRG011-19 | Poaceae | *Poa pratensis* | FCA2153-11 | Poaceae | *Poa arctica* | 100 | 848 | FALSE | rbcLa |
| SWFRG011-19 | Poaceae | *Poa pratensis* | FCA2152-11 | Poaceae | *Poa arctica* | 100 | 848 | FALSE | rbcLa |
| SWFRG011-19 | Poaceae | *Poa pratensis* | FCA2151-11 | Poaceae | *Poa arctica* | 100 | 848 | FALSE | rbcLa |
| SWFRG011-19 | Poaceae | *Poa pratensis* | FCA2149-11 | Poaceae | *Poa arctica* | 100 | 848 | FALSE | rbcLa |
| SWFRG011-19 | Poaceae | *Poa pratensis* | FCA2148-11 | Poaceae | *Poa arctica* | 100 | 848 | FALSE | rbcLa |
| SWFRG011-19 | Poaceae | *Poa pratensis* | FCA2041-11 | Poaceae | *Poa arctica* | 100 | 848 | FALSE | rbcLa |
| SWFRG011-19 | Poaceae | *Poa pratensis* | FCA2033-11 | Poaceae | *Poa pratensis* | 100 | 848 | FALSE | rbcLa |
| SWFRG011-19 | Poaceae | *Poa pratensis* | FCA1961-11 | Poaceae | *Poa arctica* | 100 | 848 | FALSE | rbcLa |
| SWFRG011-19 | Poaceae | *Poa pratensis* | FCA1538-11 | Poaceae | *Poa arctica* | 100 | 848 | FALSE | rbcLa |
| SWFRG011-19 | Poaceae | *Poa pratensis* | FCA1259-11 | Poaceae | *Poa pratensis* | 100 | 848 | FALSE | rbcLa |
| SWFRG011-19 | Poaceae | *Poa pratensis* | FCA1190-11 | Poaceae | *Alopecurus borealis* | 100 | 848 | FALSE | rbcLa |
| SWFRG011-19 | Poaceae | *Poa pratensis* | FCA1189-11 | Poaceae | *Alopecurus borealis* | 100 | 848 | FALSE | rbcLa |
| SWFRG011-19 | Poaceae | *Poa pratensis* | FCA1188-11 | Poaceae | *Alopecurus borealis* | 100 | 848 | FALSE | rbcLa |
| SWFRG011-19 | Poaceae | *Poa pratensis* | FCA1187-11 | Poaceae | *Alopecurus borealis* | 100 | 848 | FALSE | rbcLa |
| SWFRG011-19 | Poaceae | *Poa pratensis* | FCA1186-11 | Poaceae | *Alopecurus borealis* | 100 | 848 | FALSE | rbcLa |
| SWFRG011-19 | Poaceae | *Poa pratensis* | FCA1185-11 | Poaceae | *Alopecurus borealis* | 100 | 848 | FALSE | rbcLa |
| SWFRG011-19 | Poaceae | *Poa pratensis* | FCA1148-10 | Poaceae | *Poa pratensis* | 100 | 848 | FALSE | rbcLa |
| SWFRG011-19 | Poaceae | *Poa pratensis* | FCA1146-10 | Poaceae | *Poa pratensis* | 100 | 848 | FALSE | rbcLa |
| SWFRG011-19 | Poaceae | *Poa pratensis* | FCA1134-10 | Poaceae | *Poa pratensis* | 100 | 848 | FALSE | rbcLa |
| SWFRG011-19 | Poaceae | *Poa pratensis* | FCA1132-10 | Poaceae | *Poa arctica* | 100 | 848 | FALSE | rbcLa |
| SWFRG011-19 | Poaceae | *Poa pratensis* | FCA1130-10 | Poaceae | *Poa arctica* | 100 | 848 | FALSE | rbcLa |
| SWFRG011-19 | Poaceae | *Poa pratensis* | FCA1126-10 | Poaceae | *Poa arctica* | 100 | 848 | FALSE | rbcLa |
| SWFRG011-19 | Poaceae | *Poa pratensis* | FCA1124-10 | Poaceae | *Poa arctica* | 100 | 848 | FALSE | rbcLa |
| SWFRG011-19 | Poaceae | *Poa pratensis* | FCA1123-10 | Poaceae | *Poa arctica* | 100 | 848 | FALSE | rbcLa |
| SWFRG011-19 | Poaceae | *Poa pratensis* | FCA1122-10 | Poaceae | *Poa arctica* | 100 | 848 | FALSE | rbcLa |
| SWFRG011-19 | Poaceae | *Poa pratensis* | POA176-10 | Poaceae | *Poa cusickii* | 100 | 848 | FALSE | rbcLa |
| SWFRG011-19 | Poaceae | *Poa pratensis* | POA168-10 | Poaceae | *Poa pratensis* | 100 | 848 | FALSE | rbcLa |
| SWFRG011-19 | Poaceae | *Poa pratensis* | POA167-10 | Poaceae | *Poa marcida* | 100 | 848 | FALSE | rbcLa |
| SWFRG011-19 | Poaceae | *Poa pratensis* | POA166-10 | Poaceae | *Poa howellii* | 100 | 848 | FALSE | rbcLa |
| SWFRG011-19 | Poaceae | *Poa pratensis* | POA158-10 | Poaceae | *Poa macrantha* | 100 | 848 | FALSE | rbcLa |
| SWFRG011-19 | Poaceae | *Poa pratensis* | POA141-10 | Poaceae | *Poa sylvestris* | 100 | 848 | FALSE | rbcLa |
| SWFRG011-19 | Poaceae | *Poa pratensis* | POA117-10 | Poaceae | *Poa saltuensis* | 100 | 848 | FALSE | rbcLa |
| SWFRG011-19 | Poaceae | *Poa pratensis* | POA112-10 | Poaceae | *Poa pratensis* | 100 | 848 | FALSE | rbcLa |
| SWFRG011-19 | Poaceae | *Poa pratensis* | POA111-10 | Poaceae | *Poa pratensis* | 100 | 848 | FALSE | rbcLa |
| SWFRG011-19 | Poaceae | *Poa pratensis* | POA107-10 | Poaceae | *Poa alsodes* | 100 | 848 | FALSE | rbcLa |
| SWFRG011-19 | Poaceae | *Poa pratensis* | POA105-10 | Poaceae | *Poa pratensis* | 100 | 848 | FALSE | rbcLa |
| SWFRG011-19 | Poaceae | *Poa pratensis* | POA046-10 | Poaceae | *Poa alsodes* | 100 | 848 | FALSE | rbcLa |
| SWFRG011-19 | Poaceae | *Poa pratensis* | POA014-10 | Poaceae | *Poa pratensis* | 100 | 848 | FALSE | rbcLa |
| SWFRG011-19 | Poaceae | *Poa pratensis* | POA005-10 | Poaceae | *Poa pratensis* | 100 | 848 | FALSE | rbcLa |
| SWFRG011-19 | Poaceae | *Poa pratensis* | POA002-10 | Poaceae | *Poa arctica* | 100 | 848 | FALSE | rbcLa |
| SWFRG011-19 | Poaceae | *Poa pratensis* | FCA792-10 | Poaceae | *Poa pratensis* | 100 | 848 | FALSE | rbcLa |
| SWFRG011-19 | Poaceae | *Poa pratensis* | FCA790-10 | Poaceae | *Poa arctica* | 100 | 848 | FALSE | rbcLa |
| SWFRG011-19 | Poaceae | *Poa pratensis* | FCA503-10 | Poaceae | *Poa pratensis* | 100 | 848 | FALSE | rbcLa |
| SWFRG011-19 | Poaceae | *Poa pratensis* | FCA499-10 | Poaceae | *Poa pratensis* | 100 | 848 | FALSE | rbcLa |
| SWFRG011-19 | Poaceae | *Poa pratensis* | FCA495-10 | Poaceae | *Poa pratensis* | 100 | 848 | FALSE | rbcLa |
| SWFRG011-19 | Poaceae | *Poa pratensis* | FCA492-10 | Poaceae | *Poa paucispicula* | 100 | 848 | FALSE | rbcLa |
| SWFRG011-19 | Poaceae | *Poa pratensis* | FCA474-10 | Poaceae | *Poa arctica* | 100 | 848 | FALSE | rbcLa |
| SWFRG011-19 | Poaceae | *Poa pratensis* | FCA469-10 | Poaceae | *Poa arctica* | 100 | 848 | FALSE | rbcLa |
| SWFRG011-19 | Poaceae | *Poa pratensis* | FCA468-10 | Poaceae | *Poa arctica* | 100 | 848 | FALSE | rbcLa |
| SWFRG011-19 | Poaceae | *Poa pratensis* | FCA467-10 | Poaceae | *Poa arctica* | 100 | 848 | FALSE | rbcLa |
| SWFRG011-19 | Poaceae | *Poa pratensis* | FCA466-10 | Poaceae | *Poa arctica* | 100 | 848 | FALSE | rbcLa |
| SWFRG011-19 | Poaceae | *Poa pratensis* | FCA465-10 | Poaceae | *Poa arctica* | 100 | 848 | FALSE | rbcLa |
| SWFRG011-19 | Poaceae | *Poa pratensis* | FCA464-10 | Poaceae | *Poa arctica* | 100 | 848 | FALSE | rbcLa |
| SWFRG011-19 | Poaceae | *Poa pratensis* | FCA463-10 | Poaceae | *Poa arctica* | 100 | 848 | FALSE | rbcLa |
| SWFRG011-19 | Poaceae | *Poa pratensis* | FCA462-10 | Poaceae | *Poa arctica* | 100 | 848 | FALSE | rbcLa |
| SWFRG011-19 | Poaceae | *Poa pratensis* | FCA461-10 | Poaceae | *Poa arctica* | 100 | 848 | FALSE | rbcLa |
| SWFRG011-19 | Poaceae | *Poa pratensis* | FCA460-10 | Poaceae | *Poa arctica* | 100 | 848 | FALSE | rbcLa |
| SWFRG011-19 | Poaceae | *Poa pratensis* | FCA459-10 | Poaceae | *Poa arctica* | 100 | 848 | FALSE | rbcLa |
| SWFRG011-19 | Poaceae | *Poa pratensis* | FCA458-10 | Poaceae | *Poa arctica* | 100 | 848 | FALSE | rbcLa |
| SWFRG011-19 | Poaceae | *Poa pratensis* | FCA457-10 | Poaceae | *Poa arctica* | 100 | 848 | FALSE | rbcLa |
| SWFRG011-19 | Poaceae | *Poa pratensis* | FCA426-10 | Poaceae | *Alopecurus magellanicus* | 100 | 848 | FALSE | rbcLa |
| SWFRG011-19 | Poaceae | *Poa pratensis* | FCA425-10 | Poaceae | *Alopecurus borealis* | 100 | 848 | FALSE | rbcLa |
| SWFRG011-19 | Poaceae | *Poa pratensis* | GRASS1414-10 | Poaceae | *Poa wheeleri* | 100 | 848 | FALSE | rbcLa |
| SWFRG011-19 | Poaceae | *Poa pratensis* | GRASS1394-10 | Poaceae | *Poa wheeleri* | 100 | 848 | FALSE | rbcLa |
| SWFRG011-19 | Poaceae | *Poa pratensis* | GRASS1387-10 | Poaceae | *Poa pratensis* | 100 | 848 | FALSE | rbcLa |
| SWFRG011-19 | Poaceae | *Poa pratensis* | GRASS1386-10 | Poaceae | *Poa pratensis* | 100 | 848 | FALSE | rbcLa |
| SWFRG011-19 | Poaceae | *Poa pratensis* | GRASS1356-10 | Poaceae | *Poa wheeleri* | 100 | 848 | FALSE | rbcLa |
| SWFRG011-19 | Poaceae | *Poa pratensis* | GRASS1355-10 | Poaceae | *Poa howellii* | 100 | 848 | FALSE | rbcLa |
| SWFRG011-19 | Poaceae | *Poa pratensis* | GRASS1353-10 | Poaceae | *Poa wheeleri* | 100 | 848 | FALSE | rbcLa |
| SWFRG011-19 | Poaceae | *Poa pratensis* | GRASS1352-10 | Poaceae | *Poa marcida* | 100 | 848 | FALSE | rbcLa |
| SWFRG011-19 | Poaceae | *Poa pratensis* | GRASS1349-10 | Poaceae | *Poa confinis* | 100 | 848 | FALSE | rbcLa |
| SWFRG011-19 | Poaceae | *Poa pratensis* | GRASS1348-10 | Poaceae | *Poa confinis* | 100 | 848 | FALSE | rbcLa |
| SWFRG011-19 | Poaceae | *Poa pratensis* | GRASS1339-10 | Poaceae | *Poa wheeleri* | 100 | 848 | FALSE | rbcLa |
| SWFRG011-19 | Poaceae | *Poa pratensis* | FCA377-10 | Poaceae | *Alopecurus borealis* | 100 | 848 | FALSE | rbcLa |
| SWFRG011-19 | Poaceae | *Poa pratensis* | IASVF184-09 | Poaceae | *Poa arctica* | 100 | 848 | FALSE | rbcLa |
| SWFRG011-19 | Poaceae | *Poa pratensis* | IASVF160-09 | Poaceae | *Poa arctica* | 100 | 848 | FALSE | rbcLa |
| SWFRG011-19 | Poaceae | *Poa pratensis* | IASVF148-09 | Poaceae | *Alopecurus magellanicus* | 100 | 848 | FALSE | rbcLa |
| SWFRG011-19 | Poaceae | *Poa pratensis* | IASVF129-09 | Poaceae | *Poa arctica* | 100 | 848 | FALSE | rbcLa |
| SWFRG011-19 | Poaceae | *Poa pratensis* | IASVF030-09 | Poaceae | *Poa arctica* | 100 | 848 | FALSE | rbcLa |
| SWFRG011-19 | Poaceae | *Poa pratensis* | FCA256-09 | Poaceae | *Alopecurus borealis* | 100 | 848 | FALSE | rbcLa |
| SWFRG011-19 | Poaceae | *Poa pratensis* | FCA218-09 | Poaceae | *Alopecurus borealis* | 100 | 848 | FALSE | rbcLa |
| SWFRG011-19 | Poaceae | *Poa pratensis* | FCA178-09 | Poaceae | *Alopecurus borealis* | 100 | 848 | FALSE | rbcLa |
| SWFRG011-19 | Poaceae | *Poa pratensis* | FCA057-09 | Poaceae | *Poa arctica* | 100 | 848 | FALSE | rbcLa |
| SWFRG011-19 | Poaceae | *Poa pratensis* | FCA055-09 | Poaceae | *Alopecurus borealis* | 100 | 848 | FALSE | rbcLa |
| SWFRG011-19 | Poaceae | *Poa pratensis* | MKPCH388-09 | Poaceae | *Poa arctica* | 100 | 848 | FALSE | rbcLa |
| SWFRG011-19 | Poaceae | *Poa pratensis* | MKPCH068-09 | Poaceae | *Poa arctica* | 100 | 848 | FALSE | rbcLa |
| SWFRG011-19 | Poaceae | *Poa pratensis* | MKPCH067-09 | Poaceae | *Poa arctica* | 100 | 848 | FALSE | rbcLa |
| SWFRG011-19 | Poaceae | *Poa pratensis* | MKPCH022-09 | Poaceae | *Alopecurus borealis* | 100 | 848 | FALSE | rbcLa |
| SWFRG011-19 | Poaceae | *Poa pratensis* | MKPCH021-09 | Poaceae | *Alopecurus borealis* | 100 | 848 | FALSE | rbcLa |
| SWFRG011-19 | Poaceae | *Poa pratensis* | MKPCH020-09 | Poaceae | *Alopecurus borealis* | 100 | 848 | FALSE | rbcLa |
| SWFRG011-19 | Poaceae | *Poa pratensis* | PLNOR168-08 | Poaceae | *Cinna arundinacea* | 100 | 848 | FALSE | rbcLa |
| SWFRG011-19 | Poaceae | *Poa pratensis* | PLCHA240-08 | Poaceae | *Cinna arundinacea* | 100 | 848 | FALSE | rbcLa |
| SWFRG011-19 | Poaceae | *Poa pratensis* | GRASS1327-08 | Poaceae | *Poa pratensis* | 100 | 848 | FALSE | rbcLa |
| SWFRG011-19 | Poaceae | *Poa pratensis* | PLNOR061-08 | Poaceae | *Cinna arundinacea* | 100 | 848 | FALSE | rbcLa |
| SWFRG011-19 | Poaceae | *Poa pratensis* | PLCHA007-08 | Poaceae | *Poa alsodes* | 100 | 848 | FALSE | rbcLa |
| SWFRG011-19 | Poaceae | *Poa pratensis* | BPNP209-08 | Poaceae | *Poa pratensis* | 100 | 848 | FALSE | rbcLa |
| SWFRG011-19 | Poaceae | *Poa pratensis* | BPNP197-08 | Poaceae | *Poa nemoralis* | 100 | 848 | FALSE | rbcLa |
| SWFRG011-19 | Poaceae | *Poa pratensis* | GRASS1268-07 | Poaceae | *Alopecurus geniculatus* | 100 | 848 | FALSE | rbcLa |
| SWFRG011-19 | Poaceae | *Poa pratensis* | GRASS1217-07 | Poaceae | *Poa pratensis* | 100 | 848 | FALSE | rbcLa |
| SWFRG011-19 | Poaceae | *Poa pratensis* | GRASS1212-07 | Poaceae | *Poa macrantha* | 100 | 848 | FALSE | rbcLa |
| SWFRG011-19 | Poaceae | *Poa pratensis* | GRASS1191-07 | Poaceae | *Cinna latifolia* | 100 | 848 | FALSE | rbcLa |
| SWFRG011-19 | Poaceae | *Poa pratensis* | GRASS1177-07 | Poaceae | *Poa macrantha* | 100 | 848 | FALSE | rbcLa |
| SWFRG011-19 | Poaceae | *Poa pratensis* | GRASS1171-07 | Poaceae | *Poa macrantha* | 100 | 848 | FALSE | rbcLa |
| SWFRG011-19 | Poaceae | *Poa pratensis* | GRASS1125-07 | Poaceae | *Poa pratensis* | 100 | 848 | FALSE | rbcLa |
| SWFRG011-19 | Poaceae | *Poa pratensis* | GRASS1111-07 | Poaceae | *Cinna latifolia* | 100 | 848 | FALSE | rbcLa |
| SWFRG011-19 | Poaceae | *Poa pratensis* | GRASS1011-07 | Poaceae | *Alopecurus geniculatus* | 100 | 848 | FALSE | rbcLa |
| SWFRG011-19 | Poaceae | *Poa pratensis* | GRASS981-07 | Poaceae | *Poa pratensis* | 100 | 848 | FALSE | rbcLa |
| SWFRG011-19 | Poaceae | *Poa pratensis* | KSR357-07 | Poaceae | *Poa alsodes* | 100 | 848 | FALSE | rbcLa |
| SWFRG011-19 | Poaceae | *Poa pratensis* | KSR067-07 | Poaceae | *Poa pratensis* | 100 | 848 | FALSE | rbcLa |
| SWFRG011-19 | Poaceae | *Poa pratensis* | GRASS857-07 | Poaceae | *Poa macrantha* | 100 | 848 | FALSE | rbcLa |
| SWFRG011-19 | Poaceae | *Poa pratensis* | GRASS791-07 | Poaceae | *Alopecurus geniculatus* | 100 | 848 | FALSE | rbcLa |
| SWFRG011-19 | Poaceae | *Poa pratensis* | GRASS561-07 | Poaceae | *Poa pratensis* | 100 | 848 | FALSE | rbcLa |
| SWFRG011-19 | Poaceae | *Poa pratensis* | GRASS237-07 | Poaceae | *Cinna latifolia* | 100 | 848 | FALSE | rbcLa |
| SWFRG011-19 | Poaceae | *Poa pratensis* | GRASS134-07 | Poaceae | *Poa pratensis* | 100 | 848 | FALSE | rbcLa |
| SWFRG012-19 | Poaceae | *Poa pratensis* | VEMSH158-13 | Poaceae | *Poa pratensis* | 99.82 | 1031 | TRUE | rbcLa |
| SWFRG015-19 | Poaceae | *Arrhenatherum elatius* | SWFRG034-19 | Poaceae | *Trisetum flavescens* | 97.47 | 939 | FALSE | rbcLa |
| SWFRG016-19 | Poaceae | *Arrhenatherum elatius* | SDP650024-17 | Fabaceae | *Lotus corniculatus* | 98.18 | 957 | FALSE | rbcLa |
| SWFRG017-19 | Poaceae | *Dactylis glomerata* | SWFRG002-19 | Poaceae | *Dactylis glomerata* | 100 | 987 | TRUE | rbcLa |
| SWFRG018-19 | Poaceae | *Trisetum flavescens* | HIMS1693-12 | Poaceae | *Trisetum flavescens* | 100 | 931 | TRUE | rbcLa |
| SWFRG019-19 | Poaceae | *Festuca pratensis* | SWFRG010-19 | Poaceae | *Lolium perenne* | 99.44 | 974 | FALSE | rbcLa |
| SWFRG019-19 | Poaceae | *Festuca pratensis* | GRASS1202-07 | Poaceae | *Schedonorus arundinaceus* | 99.44 | 974 | FALSE | rbcLa |
| SWFRG019-19 | Poaceae | *Festuca pratensis* | GRASS1201-07 | Poaceae | *Schedonorus arundinaceus* | 99.44 | 974 | FALSE | rbcLa |
| SWFRG019-19 | Poaceae | *Festuca pratensis* | GRASS1195-07 | Poaceae | *Lolium temulentum* | 99.44 | 974 | FALSE | rbcLa |
| SWFRG019-19 | Poaceae | *Festuca pratensis* | GRASS1062-07 | Poaceae | *Schedonorus arundinaceus* | 99.44 | 974 | FALSE | rbcLa |
| SWFRG019-19 | Poaceae | *Festuca pratensis* | GRASS1034-07 | Poaceae | *Schedonorus arundinaceus* | 99.44 | 974 | FALSE | rbcLa |
| SWFRG019-19 | Poaceae | *Festuca pratensis* | GRASS1020-07 | Poaceae | *Lolium perenne* | 99.44 | 974 | FALSE | rbcLa |
| SWFRG019-19 | Poaceae | *Festuca pratensis* | GRASS986-07 | Poaceae | *Lolium perenne* | 99.44 | 974 | FALSE | rbcLa |
| SWFRG019-19 | Poaceae | *Festuca pratensis* | KSR314-07 | Poaceae | *Festuca arundinacea* | 99.44 | 974 | FALSE | rbcLa |
| SWFRG019-19 | Poaceae | *Festuca pratensis* | KSR116-07 | Poaceae | *Lolium perenne* | 99.44 | 974 | FALSE | rbcLa |
| SWFRG019-19 | Poaceae | *Festuca pratensis* | KSR077-07 | Poaceae | *Lolium arundinaceum* | 99.44 | 974 | FALSE | rbcLa |
| SWFRG019-19 | Poaceae | *Festuca pratensis* | GRASS808-07 | Poaceae | *Lolium perenne* | 99.44 | 974 | FALSE | rbcLa |
| SWFRG019-19 | Poaceae | *Festuca pratensis* | GRASS458-07 | Poaceae | *Schedonorus arundinaceus* | 99.44 | 974 | FALSE | rbcLa |
| SWFRG020-19 | Fabaceae | *Trifolium pratense* | SDP761050-18 | Fabaceae | *Trifolium pratense* | 99.44 | 977 | TRUE | rbcLa |
| SWFRG021-19 | Poaceae | *Lolium multiflorum* | SWFRG006-19 | Poaceae | *Lolium multiflorum* | 99.65 | 1042 | TRUE | rbcLa |
| SWFRG022-19 | Fabaceae | *Medicago sativa* | KSR118-07 | Fabaceae | *Medicago sativa* | 100 | 1011 | TRUE | rbcLa |
| SWFRG023-19 | Poaceae | *Festuca rubra* | SWFRG008-19 | Poaceae | *Festuca rubra* | 98.56 | 981 | TRUE | rbcLa |
| SWFRG024-19 | Fabaceae | *Lotus corniculatus* | SDP650011-17 | Fabaceae | *Lotus corniculatus* | 99.45 | 983 | TRUE | rbcLa |
| SWFRG025-19 | Poaceae | *Lolium perenne* | SWFRG010-19 | Poaceae | *Lolium perenne* | 97.7 | 821 | FALSE | rbcLa |
| SWFRG025-19 | Poaceae | *Lolium perenne* | SWFRG009-19 | Poaceae | *Lolium perenne* | 97.7 | 821 | FALSE | rbcLa |
| SWFRG025-19 | Poaceae | *Lolium perenne* | SWFRG004-19 | Poaceae | *Festuca pratensis* | 97.7 | 821 | FALSE | rbcLa |
| SWFRG025-19 | Poaceae | *Lolium perenne* | SWFRG003-19 | Poaceae | *Festuca pratensis* | 97.7 | 821 | FALSE | rbcLa |
| SWFRG025-19 | Poaceae | *Lolium perenne* | SDH3024-15 | Poaceae | *Festuca arundinacea* | 97.7 | 821 | FALSE | rbcLa |
| SWFRG025-19 | Poaceae | *Lolium perenne* | VASCB115-15 | Poaceae | *Lolium rigidum* | 97.7 | 821 | FALSE | rbcLa |
| SWFRG025-19 | Poaceae | *Lolium perenne* | SDH2439-14 | Poaceae | *Festuca pratensis* | 97.7 | 821 | FALSE | rbcLa |
| SWFRG025-19 | Poaceae | *Lolium perenne* | SERC143-14 | Poaceae | *Poaceae* | 97.7 | 821 | FALSE | rbcLa |
| SWFRG025-19 | Poaceae | *Lolium perenne* | SERC139-14 | Poaceae | *Poaceae* | 97.7 | 821 | FALSE | rbcLa |
| SWFRG025-19 | Poaceae | *Lolium perenne* | PIM239-14 | Poaceae | *Lolium arundinaceum* | 97.7 | 821 | FALSE | rbcLa |
| SWFRG025-19 | Poaceae | *Lolium perenne* | FBPL2793-13 | Poaceae | *Festuca arundinacea* | 97.7 | 821 | FALSE | rbcLa |
| SWFRG025-19 | Poaceae | *Lolium perenne* | FBPL2770-13 | Poaceae | *Lolium perenne* | 97.7 | 821 | FALSE | rbcLa |
| SWFRG025-19 | Poaceae | *Lolium perenne* | FBPL2758-13 | Poaceae | *Festuca gigantea* | 97.7 | 821 | FALSE | rbcLa |
| SWFRG025-19 | Poaceae | *Lolium perenne* | FBPL2672-13 | Poaceae | *Festuca pratensis* | 97.7 | 821 | FALSE | rbcLa |
| SWFRG025-19 | Poaceae | *Lolium perenne* | FBPL2456-13 | Poaceae | *Festuca arundinacea* | 97.7 | 821 | FALSE | rbcLa |
| SWFRG025-19 | Poaceae | *Lolium perenne* | FBPL2313-13 | Poaceae | *Festuca gigantea* | 97.7 | 821 | FALSE | rbcLa |
| SWFRG025-19 | Poaceae | *Lolium perenne* | FBPL2273-13 | Poaceae | *Lolium perenne* | 97.7 | 821 | FALSE | rbcLa |
| SWFRG025-19 | Poaceae | *Lolium perenne* | FBPL2184-13 | Poaceae | *Festuca pratensis* | 97.7 | 821 | FALSE | rbcLa |
| SWFRG025-19 | Poaceae | *Lolium perenne* | FBPL2044-13 | Poaceae | *Festuca gigantea* | 97.7 | 821 | FALSE | rbcLa |
| SWFRG025-19 | Poaceae | *Lolium perenne* | FBPL1897-13 | Poaceae | *Festuca pratensis* | 97.7 | 821 | FALSE | rbcLa |
| SWFRG025-19 | Poaceae | *Lolium perenne* | VEMSH592-13 | Poaceae | *Schedonorus arundinaceus* | 97.7 | 821 | FALSE | rbcLa |
| SWFRG025-19 | Poaceae | *Lolium perenne* | HIMS1974-12 | Poaceae | *Festuca subverticillata* | 97.7 | 821 | FALSE | rbcLa |
| SWFRG025-19 | Poaceae | *Lolium perenne* | MKTRT132-12 | Poaceae | *Lolium temulentum* | 97.7 | 821 | FALSE | rbcLa |
| SWFRG025-19 | Poaceae | *Lolium perenne* | MKTRT131-12 | Poaceae | *Lolium temulentum* | 97.7 | 821 | FALSE | rbcLa |
| SWFRG025-19 | Poaceae | *Lolium perenne* | MKTRT130-12 | Poaceae | *Lolium temulentum* | 97.7 | 821 | FALSE | rbcLa |
| SWFRG025-19 | Poaceae | *Lolium perenne* | MKTRT129-12 | Poaceae | *Lolium persicum* | 97.7 | 821 | FALSE | rbcLa |
| SWFRG025-19 | Poaceae | *Lolium perenne* | WAT047-12 | Poaceae | *Lolium arundinaceum* | 97.7 | 821 | FALSE | rbcLa |
| SWFRG025-19 | Poaceae | *Lolium perenne* | BBYUK2086-12 | Poaceae | *Schedonorus giganteus* | 97.7 | 821 | FALSE | rbcLa |
| SWFRG025-19 | Poaceae | *Lolium perenne* | BBYUK2065-12 | Poaceae | *Lolium persicum* | 97.7 | 821 | FALSE | rbcLa |
| SWFRG025-19 | Poaceae | *Lolium perenne* | GRASS1392-10 | Poaceae | *Schedonorus arundinaceus* | 97.7 | 821 | FALSE | rbcLa |
| SWFRG025-19 | Poaceae | *Lolium perenne* | PLLAM014-08 | Poaceae | *Lolium arundinaceum* | 97.7 | 821 | FALSE | rbcLa |
| SWFRG025-19 | Poaceae | *Lolium perenne* | PLCOC125-08 | Poaceae | *Lolium pratense* | 97.7 | 821 | FALSE | rbcLa |
| SWFRG025-19 | Poaceae | *Lolium perenne* | GRASS1202-07 | Poaceae | *Schedonorus arundinaceus* | 97.7 | 821 | FALSE | rbcLa |
| SWFRG025-19 | Poaceae | *Lolium perenne* | GRASS1201-07 | Poaceae | *Schedonorus arundinaceus* | 97.7 | 821 | FALSE | rbcLa |
| SWFRG025-19 | Poaceae | *Lolium perenne* | GRASS1195-07 | Poaceae | *Lolium temulentum* | 97.7 | 821 | FALSE | rbcLa |
| SWFRG025-19 | Poaceae | *Lolium perenne* | GRASS1062-07 | Poaceae | *Schedonorus arundinaceus* | 97.7 | 821 | FALSE | rbcLa |
| SWFRG025-19 | Poaceae | *Lolium perenne* | GRASS1034-07 | Poaceae | *Schedonorus arundinaceus* | 97.7 | 821 | FALSE | rbcLa |
| SWFRG025-19 | Poaceae | *Lolium perenne* | GRASS1020-07 | Poaceae | *Lolium perenne* | 97.7 | 821 | FALSE | rbcLa |
| SWFRG025-19 | Poaceae | *Lolium perenne* | GRASS986-07 | Poaceae | *Lolium perenne* | 97.7 | 821 | FALSE | rbcLa |
| SWFRG025-19 | Poaceae | *Lolium perenne* | KSR314-07 | Poaceae | *Festuca arundinacea* | 97.7 | 821 | FALSE | rbcLa |
| SWFRG025-19 | Poaceae | *Lolium perenne* | KSR116-07 | Poaceae | *Lolium perenne* | 97.7 | 821 | FALSE | rbcLa |
| SWFRG025-19 | Poaceae | *Lolium perenne* | KSR077-07 | Poaceae | *Lolium arundinaceum* | 97.7 | 821 | FALSE | rbcLa |
| SWFRG025-19 | Poaceae | *Lolium perenne* | GRASS808-07 | Poaceae | *Lolium perenne* | 97.7 | 821 | FALSE | rbcLa |
| SWFRG025-19 | Poaceae | *Lolium perenne* | GRASS458-07 | Poaceae | *Schedonorus arundinaceus* | 97.7 | 821 | FALSE | rbcLa |
| SWFRG026-19 | Fabaceae | *Trifolium repens* | SWFRG041-19 | Fabaceae | *Trifolium repens* | 99.82 | 1009 | TRUE | rbcLa |
| SWFRG028-19 | Fabaceae | *Onobrychis viciifolia* | SWFRG043-19 | Fabaceae | *Onobrychis viciifolia* | 97.45 | 935 | TRUE | rbcLa |
| SWFRG029-19 | Poaceae | *Alopecurus pratensis* | SDH3003-15 | Poaceae | *Poa bigelovii* | 99.45 | 985 | FALSE | rbcLa |
| SWFRG029-19 | Poaceae | *Alopecurus pratensis* | SDH2967-15 | Poaceae | *Poa howellii* | 99.45 | 985 | FALSE | rbcLa |
| SWFRG029-19 | Poaceae | *Alopecurus pratensis* | PCUBC840-14 | Poaceae | *Poa chaixii* | 99.45 | 985 | FALSE | rbcLa |
| SWFRG029-19 | Poaceae | *Alopecurus pratensis* | PCUBC235-14 | Poaceae | *Poa laxiflora* | 99.45 | 985 | FALSE | rbcLa |
| SWFRG029-19 | Poaceae | *Alopecurus pratensis* | PCUBC223-14 | Poaceae | *Poa laxiflora* | 99.45 | 985 | FALSE | rbcLa |
| SWFRG029-19 | Poaceae | *Alopecurus pratensis* | SDH2494-14 | Poaceae | *Poa atropurpurea* | 99.45 | 985 | FALSE | rbcLa |
| SWFRG029-19 | Poaceae | *Alopecurus pratensis* | SDH2330-14 | Poaceae | *Alopecurus pratensis* | 99.81 | 985 | FALSE | rbcLa |
| SWFRG029-19 | Poaceae | *Alopecurus pratensis* | FBPL2879-13 | Poaceae | *Poa chaixii* | 99.45 | 985 | FALSE | rbcLa |
| SWFRG029-19 | Poaceae | *Alopecurus pratensis* | FBPL2852-13 | Poaceae | *Poa chaixii* | 99.45 | 985 | FALSE | rbcLa |
| SWFRG029-19 | Poaceae | *Alopecurus pratensis* | FBPL2763-13 | Poaceae | *Cinna latifolia* | 99.45 | 985 | FALSE | rbcLa |
| SWFRG029-19 | Poaceae | *Alopecurus pratensis* | FBPL2685-13 | Poaceae | *Alopecurus arundinaceus* | 99.81 | 985 | FALSE | rbcLa |
| SWFRG029-19 | Poaceae | *Alopecurus pratensis* | FBPL2531-13 | Poaceae | *Alopecurus arundinaceus* | 99.81 | 985 | FALSE | rbcLa |
| SWFRG029-19 | Poaceae | *Alopecurus pratensis* | FBPL2448-13 | Poaceae | *Alopecurus arundinaceus* | 99.81 | 985 | FALSE | rbcLa |
| SWFRG029-19 | Poaceae | *Alopecurus pratensis* | FBPL2387-13 | Poaceae | *Cinna latifolia* | 99.45 | 985 | FALSE | rbcLa |
| SWFRG029-19 | Poaceae | *Alopecurus pratensis* | FBPL2350-13 | Poaceae | *Alopecurus geniculatus* | 99.45 | 985 | FALSE | rbcLa |
| SWFRG029-19 | Poaceae | *Alopecurus pratensis* | FBPL2267-13 | Poaceae | *Alopecurus geniculatus* | 99.45 | 985 | FALSE | rbcLa |
| SWFRG029-19 | Poaceae | *Alopecurus pratensis* | FBPL2187-13 | Poaceae | *Alopecurus geniculatus* | 99.45 | 985 | FALSE | rbcLa |
| SWFRG029-19 | Poaceae | *Alopecurus pratensis* | FBPL2183-13 | Poaceae | *Alopecurus pratensis* | 99.81 | 985 | FALSE | rbcLa |
| SWFRG029-19 | Poaceae | *Alopecurus pratensis* | FBPL2048-13 | Poaceae | *Cinna latifolia* | 99.45 | 985 | FALSE | rbcLa |
| SWFRG029-19 | Poaceae | *Alopecurus pratensis* | FBPL2001-13 | Poaceae | *Alopecurus pratensis* | 99.81 | 985 | FALSE | rbcLa |
| SWFRG029-19 | Poaceae | *Alopecurus pratensis* | FBPL1989-13 | Poaceae | *Poa remota* | 99.45 | 985 | FALSE | rbcLa |
| SWFRG029-19 | Poaceae | *Alopecurus pratensis* | FBPL1945-13 | Poaceae | *Poa chaixii* | 99.45 | 985 | FALSE | rbcLa |
| SWFRG029-19 | Poaceae | *Alopecurus pratensis* | FBPL1916-13 | Poaceae | *Alopecurus arundinaceus* | 99.81 | 985 | FALSE | rbcLa |
| SWFRG029-19 | Poaceae | *Alopecurus pratensis* | FBPL1914-13 | Poaceae | *Alopecurus arundinaceus* | 99.81 | 985 | FALSE | rbcLa |
| SWFRG029-19 | Poaceae | *Alopecurus pratensis* | FBPL1854-13 | Poaceae | *Poa remota* | 99.45 | 985 | FALSE | rbcLa |
| SWFRG029-19 | Poaceae | *Alopecurus pratensis* | FBPL1519-13 | Poaceae | *Poa remota* | 99.45 | 985 | FALSE | rbcLa |
| SWFRG029-19 | Poaceae | *Alopecurus pratensis* | FBPL1449-13 | Poaceae | *Alopecurus aequalis* | 99.45 | 985 | FALSE | rbcLa |
| SWFRG029-19 | Poaceae | *Alopecurus pratensis* | FBPL1295-13 | Poaceae | *Alopecurus aequalis* | 99.45 | 985 | FALSE | rbcLa |
| SWFRG029-19 | Poaceae | *Alopecurus pratensis* | WABLK276-13 | Poaceae | *Cinna latifolia* | 99.45 | 985 | FALSE | rbcLa |
| SWFRG029-19 | Poaceae | *Alopecurus pratensis* | VPSBC476-13 | Poaceae | *Poa marcida* | 99.45 | 985 | FALSE | rbcLa |
| SWFRG029-19 | Poaceae | *Alopecurus pratensis* | GRAFW1907-13 | Poaceae | *Alopecurus borealis* | 99.45 | 985 | FALSE | rbcLa |
| SWFRG029-19 | Poaceae | *Alopecurus pratensis* | REDNO049-12 | Poaceae | *Cinna latifolia* | 99.45 | 985 | FALSE | rbcLa |
| SWFRG029-19 | Poaceae | *Alopecurus pratensis* | HIMS1861-12 | Poaceae | *Poa fendleriana* | 99.45 | 985 | FALSE | rbcLa |
| SWFRG029-19 | Poaceae | *Alopecurus pratensis* | HIMS1645-12 | Poaceae | *Poa nervosa* | 99.45 | 985 | FALSE | rbcLa |
| SWFRG029-19 | Poaceae | *Alopecurus pratensis* | MSTH046-12 | Poaceae | *Poa saltuensis* | 99.45 | 985 | FALSE | rbcLa |
| SWFRG029-19 | Poaceae | *Alopecurus pratensis* | WAT227-12 | Poaceae | *Alopecurus pratensis* | 99.81 | 985 | FALSE | rbcLa |
| SWFRG029-19 | Poaceae | *Alopecurus pratensis* | BBYUK2092-12 | Poaceae | *Poa macrantha* | 99.45 | 985 | FALSE | rbcLa |
| SWFRG029-19 | Poaceae | *Alopecurus pratensis* | BBYUK2081-12 | Poaceae | *Poa porsildii* | 99.45 | 985 | FALSE | rbcLa |
| SWFRG029-19 | Poaceae | *Alopecurus pratensis* | BBYUK2080-12 | Poaceae | *Poa porsildii* | 99.45 | 985 | FALSE | rbcLa |
| SWFRG029-19 | Poaceae | *Alopecurus pratensis* | BBYUK2078-12 | Poaceae | *Poa cusickii* | 99.45 | 985 | FALSE | rbcLa |
| SWFRG029-19 | Poaceae | *Alopecurus pratensis* | FOND005-12 | Poaceae | *Alopecurus arundinaceus* | 99.81 | 985 | FALSE | rbcLa |
| SWFRG029-19 | Poaceae | *Alopecurus pratensis* | PLPAR012-11 | Poaceae | *Cinna latifolia* | 99.45 | 985 | FALSE | rbcLa |
| SWFRG029-19 | Poaceae | *Alopecurus pratensis* | PLKEN290-11 | Poaceae | *Poa pratensis* | 99.45 | 985 | FALSE | rbcLa |
| SWFRG029-19 | Poaceae | *Alopecurus pratensis* | PLCOC134-11 | Poaceae | *Cinna latifolia* | 99.45 | 985 | FALSE | rbcLa |
| SWFRG029-19 | Poaceae | *Alopecurus pratensis* | PLTHU150-11 | Poaceae | *Poa pratensis* | 99.45 | 985 | FALSE | rbcLa |
| SWFRG029-19 | Poaceae | *Alopecurus pratensis* | FCA2986-11 | Poaceae | *Poa paucispicula* | 99.45 | 985 | FALSE | rbcLa |
| SWFRG029-19 | Poaceae | *Alopecurus pratensis* | FCA2985-11 | Poaceae | *Poa paucispicula* | 99.45 | 985 | FALSE | rbcLa |
| SWFRG029-19 | Poaceae | *Alopecurus pratensis* | FCA2984-11 | Poaceae | *Poa paucispicula* | 99.45 | 985 | FALSE | rbcLa |
| SWFRG029-19 | Poaceae | *Alopecurus pratensis* | FCA2844-11 | Poaceae | *Alopecurus borealis* | 99.45 | 985 | FALSE | rbcLa |
| SWFRG029-19 | Poaceae | *Alopecurus pratensis* | FCA1190-11 | Poaceae | *Alopecurus borealis* | 99.45 | 985 | FALSE | rbcLa |
| SWFRG029-19 | Poaceae | *Alopecurus pratensis* | FCA1189-11 | Poaceae | *Alopecurus borealis* | 99.45 | 985 | FALSE | rbcLa |
| SWFRG029-19 | Poaceae | *Alopecurus pratensis* | FCA1188-11 | Poaceae | *Alopecurus borealis* | 99.45 | 985 | FALSE | rbcLa |
| SWFRG029-19 | Poaceae | *Alopecurus pratensis* | FCA1187-11 | Poaceae | *Alopecurus borealis* | 99.45 | 985 | FALSE | rbcLa |
| SWFRG029-19 | Poaceae | *Alopecurus pratensis* | FCA1186-11 | Poaceae | *Alopecurus borealis* | 99.45 | 985 | FALSE | rbcLa |
| SWFRG029-19 | Poaceae | *Alopecurus pratensis* | FCA1185-11 | Poaceae | *Alopecurus borealis* | 99.45 | 985 | FALSE | rbcLa |
| SWFRG029-19 | Poaceae | *Alopecurus pratensis* | POA176-10 | Poaceae | *Poa cusickii* | 99.45 | 985 | FALSE | rbcLa |
| SWFRG029-19 | Poaceae | *Alopecurus pratensis* | POA167-10 | Poaceae | *Poa marcida* | 99.45 | 985 | FALSE | rbcLa |
| SWFRG029-19 | Poaceae | *Alopecurus pratensis* | POA166-10 | Poaceae | *Poa howellii* | 99.45 | 985 | FALSE | rbcLa |
| SWFRG029-19 | Poaceae | *Alopecurus pratensis* | POA158-10 | Poaceae | *Poa macrantha* | 99.45 | 985 | FALSE | rbcLa |
| SWFRG029-19 | Poaceae | *Alopecurus pratensis* | POA141-10 | Poaceae | *Poa sylvestris* | 99.45 | 985 | FALSE | rbcLa |
| SWFRG029-19 | Poaceae | *Alopecurus pratensis* | POA117-10 | Poaceae | *Poa saltuensis* | 99.45 | 985 | FALSE | rbcLa |
| SWFRG029-19 | Poaceae | *Alopecurus pratensis* | POA107-10 | Poaceae | *Poa alsodes* | 99.45 | 985 | FALSE | rbcLa |
| SWFRG029-19 | Poaceae | *Alopecurus pratensis* | POA046-10 | Poaceae | *Poa alsodes* | 99.45 | 985 | FALSE | rbcLa |
| SWFRG029-19 | Poaceae | *Alopecurus pratensis* | FCA492-10 | Poaceae | *Poa paucispicula* | 99.45 | 985 | FALSE | rbcLa |
| SWFRG029-19 | Poaceae | *Alopecurus pratensis* | FCA426-10 | Poaceae | *Alopecurus magellanicus* | 99.45 | 985 | FALSE | rbcLa |
| SWFRG029-19 | Poaceae | *Alopecurus pratensis* | FCA425-10 | Poaceae | *Alopecurus borealis* | 99.45 | 985 | FALSE | rbcLa |
| SWFRG029-19 | Poaceae | *Alopecurus pratensis* | GRASS1414-10 | Poaceae | *Poa wheeleri* | 99.45 | 985 | FALSE | rbcLa |
| SWFRG029-19 | Poaceae | *Alopecurus pratensis* | GRASS1411-10 | Poaceae | *Alopecurus pratensis* | 99.81 | 985 | FALSE | rbcLa |
| SWFRG029-19 | Poaceae | *Alopecurus pratensis* | GRASS1407-10 | Poaceae | *Alopecurus pratensis* | 99.81 | 985 | FALSE | rbcLa |
| SWFRG029-19 | Poaceae | *Alopecurus pratensis* | GRASS1394-10 | Poaceae | *Poa wheeleri* | 99.45 | 985 | FALSE | rbcLa |
| SWFRG029-19 | Poaceae | *Alopecurus pratensis* | GRASS1393-10 | Poaceae | *Alopecurus pratensis* | 99.81 | 985 | FALSE | rbcLa |
| SWFRG029-19 | Poaceae | *Alopecurus pratensis* | GRASS1377-10 | Poaceae | *Alopecurus pratensis* | 99.81 | 985 | FALSE | rbcLa |
| SWFRG029-19 | Poaceae | *Alopecurus pratensis* | GRASS1356-10 | Poaceae | *Poa wheeleri* | 99.45 | 985 | FALSE | rbcLa |
| SWFRG029-19 | Poaceae | *Alopecurus pratensis* | GRASS1355-10 | Poaceae | *Poa howellii* | 99.45 | 985 | FALSE | rbcLa |
| SWFRG029-19 | Poaceae | *Alopecurus pratensis* | GRASS1353-10 | Poaceae | *Poa wheeleri* | 99.45 | 985 | FALSE | rbcLa |
| SWFRG029-19 | Poaceae | *Alopecurus pratensis* | GRASS1352-10 | Poaceae | *Poa marcida* | 99.45 | 985 | FALSE | rbcLa |
| SWFRG029-19 | Poaceae | *Alopecurus pratensis* | GRASS1349-10 | Poaceae | *Poa confinis* | 99.45 | 985 | FALSE | rbcLa |
| SWFRG029-19 | Poaceae | *Alopecurus pratensis* | GRASS1348-10 | Poaceae | *Poa confinis* | 99.45 | 985 | FALSE | rbcLa |
| SWFRG029-19 | Poaceae | *Alopecurus pratensis* | GRASS1339-10 | Poaceae | *Poa wheeleri* | 99.45 | 985 | FALSE | rbcLa |
| SWFRG029-19 | Poaceae | *Alopecurus pratensis* | FCA377-10 | Poaceae | *Alopecurus borealis* | 99.45 | 985 | FALSE | rbcLa |
| SWFRG029-19 | Poaceae | *Alopecurus pratensis* | IASVF148-09 | Poaceae | *Alopecurus magellanicus* | 99.45 | 985 | FALSE | rbcLa |
| SWFRG029-19 | Poaceae | *Alopecurus pratensis* | FCA256-09 | Poaceae | *Alopecurus borealis* | 99.45 | 985 | FALSE | rbcLa |
| SWFRG029-19 | Poaceae | *Alopecurus pratensis* | FCA218-09 | Poaceae | *Alopecurus borealis* | 99.45 | 985 | FALSE | rbcLa |
| SWFRG029-19 | Poaceae | *Alopecurus pratensis* | FCA055-09 | Poaceae | *Alopecurus borealis* | 99.45 | 985 | FALSE | rbcLa |
| SWFRG029-19 | Poaceae | *Alopecurus pratensis* | MKPCH022-09 | Poaceae | *Alopecurus borealis* | 99.45 | 985 | FALSE | rbcLa |
| SWFRG029-19 | Poaceae | *Alopecurus pratensis* | MKPCH021-09 | Poaceae | *Alopecurus borealis* | 99.45 | 985 | FALSE | rbcLa |
| SWFRG029-19 | Poaceae | *Alopecurus pratensis* | MKPCH020-09 | Poaceae | *Alopecurus borealis* | 99.45 | 985 | FALSE | rbcLa |
| SWFRG029-19 | Poaceae | *Alopecurus pratensis* | PLCHA007-08 | Poaceae | *Poa alsodes* | 99.45 | 985 | FALSE | rbcLa |
| SWFRG029-19 | Poaceae | *Alopecurus pratensis* | GRASS1268-07 | Poaceae | *Alopecurus geniculatus* | 99.45 | 985 | FALSE | rbcLa |
| SWFRG029-19 | Poaceae | *Alopecurus pratensis* | GRASS1212-07 | Poaceae | *Poa macrantha* | 99.45 | 985 | FALSE | rbcLa |
| SWFRG029-19 | Poaceae | *Alopecurus pratensis* | GRASS1191-07 | Poaceae | *Cinna latifolia* | 99.45 | 985 | FALSE | rbcLa |
| SWFRG029-19 | Poaceae | *Alopecurus pratensis* | GRASS1177-07 | Poaceae | *Poa macrantha* | 99.45 | 985 | FALSE | rbcLa |
| SWFRG029-19 | Poaceae | *Alopecurus pratensis* | GRASS1171-07 | Poaceae | *Poa macrantha* | 99.45 | 985 | FALSE | rbcLa |
| SWFRG029-19 | Poaceae | *Alopecurus pratensis* | GRASS1111-07 | Poaceae | *Cinna latifolia* | 99.45 | 985 | FALSE | rbcLa |
| SWFRG029-19 | Poaceae | *Alopecurus pratensis* | GRASS1016-07 | Poaceae | *Alopecurus pratensis* | 99.81 | 985 | FALSE | rbcLa |
| SWFRG029-19 | Poaceae | *Alopecurus pratensis* | GRASS1011-07 | Poaceae | *Alopecurus geniculatus* | 99.45 | 985 | FALSE | rbcLa |
| SWFRG029-19 | Poaceae | *Alopecurus pratensis* | KSR357-07 | Poaceae | *Poa alsodes* | 99.45 | 985 | FALSE | rbcLa |
| SWFRG029-19 | Poaceae | *Alopecurus pratensis* | GRASS857-07 | Poaceae | *Poa macrantha* | 99.45 | 985 | FALSE | rbcLa |
| SWFRG029-19 | Poaceae | *Alopecurus pratensis* | GRASS791-07 | Poaceae | *Alopecurus geniculatus* | 99.45 | 985 | FALSE | rbcLa |
| SWFRG029-19 | Poaceae | *Alopecurus pratensis* | GRASS237-07 | Poaceae | *Cinna latifolia* | 99.45 | 985 | FALSE | rbcLa |
| SWFRG030-19 | Poaceae | *Cynosurus cristatus* | SWFRG045-19 | Poaceae | *Cynosurus cristatus* | 99.81 | 990 | TRUE | rbcLa |
| SWFRG032-19 | Poaceae | *Phleum pratense* | SWFRG016-19 | Poaceae | *Arrhenatherum elatius* | 98.1 | 917 | FALSE | rbcLa |
| SWFRG033-19 | Poaceae | *Trisetum flavescens* | VEMSH209-13 | Poaceae | *Trisetum spicatum* | 99.47 | 1035 | FALSE | rbcLa |
| SWFRG034-19 | Poaceae | *Trisetum flavescens* | SWFRG033-19 | Poaceae | *Trisetum flavescens* | 99.82 | 1002 | TRUE | rbcLa |
| SWFRG035-19 | Fabaceae | *Trifolium pratense* | SDP761050-18 | Fabaceae | *Trifolium pratense* | 99.64 | 1002 | TRUE | rbcLa |
| SWFRG036-19 | Fabaceae | *Trifolium pratense* | SDP761061-18 | Fabaceae | *Trifolium pratense* | 99.8 | 933 | FALSE | rbcLa |
| SWFRG036-19 | Fabaceae | *Trifolium pratense* | SDP761050-18 | Fabaceae | *Trifolium pratense* | 99.8 | 933 | FALSE | rbcLa |
| SWFRG036-19 | Fabaceae | *Trifolium pratense* | SDP761033-18 | Fabaceae | *Trifolium pratense* | 99.8 | 933 | FALSE | rbcLa |
| SWFRG036-19 | Fabaceae | *Trifolium pratense* | SDP761004-18 | Fabaceae | *Trifolium pratense* | 99.8 | 933 | FALSE | rbcLa |
| SWFRG036-19 | Fabaceae | *Trifolium pratense* | VPSBC1502-14 | Fabaceae | *Trifolium pratense* | 99.8 | 933 | FALSE | rbcLa |
| SWFRG036-19 | Fabaceae | *Trifolium pratense* | VPSBC1474-14 | Fabaceae | *Trifolium pratense* | 99.8 | 933 | FALSE | rbcLa |
| SWFRG036-19 | Fabaceae | *Trifolium pratense* | VPSBC1402-14 | Fabaceae | *Trifolium pratense* | 99.8 | 933 | FALSE | rbcLa |
| SWFRG036-19 | Fabaceae | *Trifolium pratense* | PIM168-14 | Fabaceae | *Trifolium pratense* | 99.8 | 933 | FALSE | rbcLa |
| SWFRG036-19 | Fabaceae | *Trifolium pratense* | VPSBC994-13 | Fabaceae | *Trifolium pratense* | 99.8 | 933 | FALSE | rbcLa |
| SWFRG036-19 | Fabaceae | *Trifolium pratense* | WABLK187-13 | Fabaceae | *Trifolium pratense* | 99.8 | 933 | FALSE | rbcLa |
| SWFRG036-19 | Fabaceae | *Trifolium pratense* | HIMS2242-12 | Fabaceae | *Trifolium repens* | 99.8 | 933 | FALSE | rbcLa |
| SWFRG036-19 | Fabaceae | *Trifolium pratense* | HIMS2090-12 | Fabaceae | *Trifolium pratense* | 99.8 | 933 | FALSE | rbcLa |
| SWFRG036-19 | Fabaceae | *Trifolium pratense* | HIMS2089-12 | Fabaceae | *Trifolium pratense* | 99.8 | 933 | FALSE | rbcLa |
| SWFRG036-19 | Fabaceae | *Trifolium pratense* | HIMS2088-12 | Fabaceae | *Trifolium pratense* | 99.8 | 933 | FALSE | rbcLa |
| SWFRG036-19 | Fabaceae | *Trifolium pratense* | WAT114-12 | Fabaceae | *Trifolium pratense* | 99.8 | 933 | FALSE | rbcLa |
| SWFRG036-19 | Fabaceae | *Trifolium pratense* | WAT087-12 | Fabaceae | *Trifolium pratense* | 99.8 | 933 | FALSE | rbcLa |
| SWFRG036-19 | Fabaceae | *Trifolium pratense* | PLKEN314-11 | Fabaceae | *Trifolium pratense* | 99.8 | 933 | FALSE | rbcLa |
| SWFRG036-19 | Fabaceae | *Trifolium pratense* | PLKEN099-10 | Fabaceae | *Trifolium pratense* | 99.8 | 933 | FALSE | rbcLa |
| SWFRG037-19 | Fabaceae | *Medicago sativa* | SWFRG022-19 | Fabaceae | *Medicago sativa* | 99.81 | 957 | TRUE | rbcLa |
| SWFRG038-19 | Fabaceae | *Medicago sativa* | KSR118-07 | Fabaceae | *Medicago sativa* | 99.44 | 977 | TRUE | rbcLa |
| SWFRG039-19 | Fabaceae | *Lotus corniculatus* | SDP650024-17 | Fabaceae | *Lotus corniculatus* | 98.89 | 957 | TRUE | rbcLa |
| SWFRG039-19 | Fabaceae | *Lotus corniculatus* | SDP650011-17 | Fabaceae | *Lotus corniculatus* | 98.89 | 957 | TRUE | rbcLa |
| SWFRG040-19 | Fabaceae | *Lotus corniculatus* | SDP650024-17 | Fabaceae | *Lotus corniculatus* | 99.8 | 902 | TRUE | rbcLa |
| SWFRG040-19 | Fabaceae | *Lotus corniculatus* | KSR068-07 | Fabaceae | *Lotus corniculatus* | 99.8 | 902 | TRUE | rbcLa |
| SWFRG041-19 | Fabaceae | *Trifolium repens* | SWFRG042-19 | Fabaceae | *Trifolium repens* | 99.65 | 1037 | TRUE | rbcLa |
| SWFRG042-19 | Fabaceae | *Trifolium repens* | SWFRG041-19 | Fabaceae | *Trifolium repens* | 99.65 | 1037 | TRUE | rbcLa |
| SWFRG043-19 | Fabaceae | *Onobrychis viciifolia* | VPSBC974-13 | Fabaceae | *Onobrychis viciifolia* | 99.82 | 1014 | TRUE | rbcLa |
| SWFRG043-19 | Fabaceae | *Onobrychis viciifolia* | BBYUK1100-12 | Fabaceae | *Onobrychis viciifolia* | 99.82 | 1014 | TRUE | rbcLa |
| SWFRG044-19 | Fabaceae | *Onobrychis viciifolia* | SWFRG028-19 | Fabaceae | *Onobrychis viciifolia* | 96.9 | 913 | TRUE | rbcLa |
| SWFRG045-19 | Poaceae | *Cynosurus cristatus* | VEMSH204-13 | Poaceae | *Festuca subverticillata* | 99.12 | 1026 | FALSE | rbcLa |
| SWFRG046-19 | Poaceae | *Cynosurus cristatus* | SWFRG045-19 | Poaceae | *Cynosurus cristatus* | 99.81 | 972 | TRUE | rbcLa |
| SWFRG047-19 | Poaceae | *Phleum pratense* | VEMSH223-13 | Poaceae | *Phleum pratense* | 99.82 | 1046 | TRUE | rbcLa |
| SWFRG001-19 | Poaceae | *Dactylis glomerata* | PLGE143-13 | Poaceae | *Dactylis glomerata* | 94.07 | 939 | TRUE | matK |
| SWFRG002-19 | Poaceae | *Dactylis glomerata* | GBVD3276-11 | Poaceae | *Dactylis glomerata* | 98.15 | 1535 | TRUE | matK |
| SWFRG003-19 | Poaceae | *Festuca pratensis* | SWFRG004-19 | Poaceae | *Festuca pratensis* | 98.79 | 1027 | TRUE | matK |
| SWFRG004-19 | Poaceae | *Festuca pratensis* | GBVX1974-13 | Poaceae | *Festuca pratensis* | 99.89 | 1605 | TRUE | matK |
| SWFRG004-19 | Poaceae | *Festuca pratensis* | GBVW3313-13 | Poaceae | *Festuca pratensis* | 99.89 | 1605 | TRUE | matK |
| SWFRG005-19 | Poaceae | *Lolium multiflorum* | SWFRG004-19 | Poaceae | *Festuca pratensis* | 99.66 | 1594 | FALSE | matK |
| SWFRG005-19 | Poaceae | *Lolium multiflorum* | GBVX1975-13 | Poaceae | *Lolium multiflorum* | 99.66 | 1594 | FALSE | matK |
| SWFRG005-19 | Poaceae | *Lolium multiflorum* | GBVW3314-13 | Poaceae | *Lolium multiflorum* | 99.66 | 1594 | FALSE | matK |
| SWFRG005-19 | Poaceae | *Lolium multiflorum* | GBVR3374-13 | Poaceae | *Lolium multiflorum* | 99.66 | 1594 | FALSE | matK |
| SWFRG005-19 | Poaceae | *Lolium multiflorum* | GBVD3994-11 | Poaceae | *Lolium rigidum* | 99.66 | 1594 | FALSE | matK |
| SWFRG006-19 | Poaceae | *Lolium multiflorum* | GBVX1975-13 | Poaceae | *Lolium multiflorum* | 99.77 | 1600 | FALSE | matK |
| SWFRG006-19 | Poaceae | *Lolium multiflorum* | GBVW3314-13 | Poaceae | *Lolium multiflorum* | 99.77 | 1600 | FALSE | matK |
| SWFRG006-19 | Poaceae | *Lolium multiflorum* | GBVR3374-13 | Poaceae | *Lolium multiflorum* | 99.77 | 1600 | FALSE | matK |
| SWFRG006-19 | Poaceae | *Lolium multiflorum* | GBVD3994-11 | Poaceae | *Lolium rigidum* | 99.77 | 1600 | FALSE | matK |
| SWFRG007-19 | Poaceae | *Festuca rubra* | GBVX1973-13 | Poaceae | *Festuca ovina* | 99.89 | 1600 | FALSE | matK |
| SWFRG007-19 | Poaceae | *Festuca rubra* | GBVW3312-13 | Poaceae | *Festuca ovina* | 99.89 | 1600 | FALSE | matK |
| SWFRG008-19 | Poaceae | *Festuca rubra* | GBVR3368-13 | Poaceae | *Festuca petraea* | 99.54 | 1583 | FALSE | matK |
| SWFRG009-19 | Poaceae | *Lolium perenne* | GBVX1861-13 | Poaceae | *Lolium perenne* | 99.77 | 1600 | TRUE | matK |
| SWFRG009-19 | Poaceae | *Lolium perenne* | GBVR1869-13 | Poaceae | *Lolium perenne* | 99.77 | 1600 | TRUE | matK |
| SWFRG009-19 | Poaceae | *Lolium perenne* | GBVD3989-11 | Poaceae | *Lolium perenne* | 99.77 | 1600 | TRUE | matK |
| SWFRG010-19 | Poaceae | *Lolium perenne* | GBVX1861-13 | Poaceae | *Lolium perenne* | 99.38 | 874 | TRUE | matK |
| SWFRG010-19 | Poaceae | *Lolium perenne* | GBVR1869-13 | Poaceae | *Lolium perenne* | 99.38 | 874 | TRUE | matK |
| SWFRG010-19 | Poaceae | *Lolium perenne* | GBVD3989-11 | Poaceae | *Lolium perenne* | 99.38 | 874 | TRUE | matK |
| SWFRG011-19 | Poaceae | *Poa pratensis* | SWFRG012-19 | Poaceae | *Poa pratensis* | 98.73 | 1539 | FALSE | matK |
| SWFRG011-19 | Poaceae | *Poa pratensis* | GBVR1837-13 | Poaceae | *Hyalopoa lanatiflora* | 98.73 | 1539 | FALSE | matK |
| SWFRG012-19 | Poaceae | *Poa pratensis* | GBVR1837-13 | Poaceae | *Hyalopoa lanatiflora* | 99.77 | 1592 | FALSE | matK |
| SWFRG013-19 | Poaceae | *Alopecurus pratensis* | GBVS364-13 | Poaceae | *Cynosurus cristatus* | 99.64 | 1531 | FALSE | matK |
| SWFRG014-19 | Poaceae | *Alopecurus pratensis* | SWFRG031-19 | Poaceae | *Arrhenatherum elatius* | 100 | 1225 | FALSE | matK |
| SWFRG014-19 | Poaceae | *Alopecurus pratensis* | SWFRG025-19 | Poaceae | *Lolium perenne* | 100 | 1225 | FALSE | matK |
| SWFRG014-19 | Poaceae | *Alopecurus pratensis* | FBPL1386-13 | Poaceae | *Cynosurus cristatus* | 100 | 1225 | FALSE | matK |
| SWFRG014-19 | Poaceae | *Alopecurus pratensis* | GBVS364-13 | Poaceae | *Cynosurus cristatus* | 100 | 1225 | FALSE | matK |
| SWFRG014-19 | Poaceae | *Alopecurus pratensis* | GRASS1174-07 | Poaceae | *Cynosurus cristatus* | 100 | 1225 | FALSE | matK |
| SWFRG014-19 | Poaceae | *Alopecurus pratensis* | GRASS1038-07 | Poaceae | *Cynosurus cristatus* | 100 | 1225 | FALSE | matK |
| SWFRG014-19 | Poaceae | *Alopecurus pratensis* | GRASS964-07 | Poaceae | *Cynosurus cristatus* | 100 | 1225 | FALSE | matK |
| SWFRG015-19 | Poaceae | *Arrhenatherum elatius* | GBVR1776-13 | Poaceae | *Arrhenatherum elatius* | 97.72 | 747 | TRUE | matK |
| SWFRG015-19 | Poaceae | *Arrhenatherum elatius* | GBVD2878-11 | Poaceae | *Arrhenatherum elatius* | 97.72 | 747 | TRUE | matK |
| SWFRG015-19 | Poaceae | *Arrhenatherum elatius* | GRASS1063-07 | Poaceae | *Arrhenatherum elatius* | 97.72 | 747 | TRUE | matK |
| SWFRG015-19 | Poaceae | *Arrhenatherum elatius* | GRASS1024-07 | Poaceae | *Arrhenatherum elatius* | 97.72 | 747 | TRUE | matK |
| SWFRG016-19 | Poaceae | *Arrhenatherum elatius* | GBVS364-13 | Poaceae | *Cynosurus cristatus* | 95.11 | 942 | FALSE | matK |
| SWFRG017-19 | Poaceae | *Dactylis glomerata* | GBVD3276-11 | Poaceae | *Dactylis glomerata* | 98.04 | 1531 | TRUE | matK |
| SWFRG018-19 | Poaceae | *Trisetum flavescens* | SWFRG034-19 | Poaceae | *Trisetum flavescens* | 98.5 | 1531 | TRUE | matK |
| SWFRG019-19 | Poaceae | *Festuca pratensis* | GBVX1975-13 | Poaceae | *Lolium multiflorum* | 99.49 | 1074 | FALSE | matK |
| SWFRG019-19 | Poaceae | *Festuca pratensis* | GBVX1974-13 | Poaceae | *Festuca pratensis* | 99.49 | 1074 | FALSE | matK |
| SWFRG019-19 | Poaceae | *Festuca pratensis* | GBVW3314-13 | Poaceae | *Lolium multiflorum* | 99.49 | 1074 | FALSE | matK |
| SWFRG019-19 | Poaceae | *Festuca pratensis* | GBVW3313-13 | Poaceae | *Festuca pratensis* | 99.49 | 1074 | FALSE | matK |
| SWFRG019-19 | Poaceae | *Festuca pratensis* | GBVW1902-13 | Poaceae | *Lolium perenne* | 99.49 | 1074 | FALSE | matK |
| SWFRG019-19 | Poaceae | *Festuca pratensis* | GBVR3374-13 | Poaceae | *Lolium multiflorum* | 99.49 | 1074 | FALSE | matK |
| SWFRG019-19 | Poaceae | *Festuca pratensis* | PLGE092-13 | Poaceae | *Lolium multiflorum* | 99.49 | 1074 | FALSE | matK |
| SWFRG019-19 | Poaceae | *Festuca pratensis* | GBVD3994-11 | Poaceae | *Lolium rigidum* | 99.49 | 1074 | FALSE | matK |
| SWFRG019-19 | Poaceae | *Festuca pratensis* | GRASS977-07 | Poaceae | *Lolium multiflorum* | 99.49 | 1074 | FALSE | matK |
| SWFRG019-19 | Poaceae | *Festuca pratensis* | KSR314-07 | Poaceae | *Festuca arundinacea* | 99.49 | 1074 | FALSE | matK |
| SWFRG021-19 | Poaceae | *Lolium multiflorum* | GBVX1975-13 | Poaceae | *Lolium multiflorum* | 96.46 | 963 | FALSE | matK |
| SWFRG021-19 | Poaceae | *Lolium multiflorum* | GBVW3314-13 | Poaceae | *Lolium multiflorum* | 96.46 | 963 | FALSE | matK |
| SWFRG021-19 | Poaceae | *Lolium multiflorum* | GBVR3374-13 | Poaceae | *Lolium multiflorum* | 96.46 | 963 | FALSE | matK |
| SWFRG021-19 | Poaceae | *Lolium multiflorum* | GBVD3994-11 | Poaceae | *Lolium rigidum* | 96.46 | 963 | FALSE | matK |
| SWFRG022-19 | Fabaceae | *Medicago sativa* | FOND125-12 | Fabaceae | *Medicago sativa* | 98.78 | 728 | TRUE | matK |
| SWFRG022-19 | Fabaceae | *Medicago sativa* | FOND124-12 | Fabaceae | *Medicago sativa* | 98.78 | 728 | TRUE | matK |
| SWFRG022-19 | Fabaceae | *Medicago sativa* | GBVO1763-11 | Fabaceae | *Medicago sativa* | 98.78 | 728 | TRUE | matK |
| SWFRG022-19 | Fabaceae | *Medicago sativa* | PLCHA137-08 | Fabaceae | *Medicago sativa* | 98.78 | 728 | TRUE | matK |
| SWFRG022-19 | Fabaceae | *Medicago sativa* | KSR118-07 | Fabaceae | *Medicago sativa* | 98.78 | 728 | TRUE | matK |
| SWFRG023-19 | Poaceae | *Festuca rubra* | SWFRG008-19 | Poaceae | *Festuca rubra* | 90.54 | 688 | TRUE | matK |
| SWFRG024-19 | Fabaceae | *Lotus corniculatus* | SWFRG040-19 | Fabaceae | *Lotus corniculatus* | 90.46 | 503 | FALSE | matK |
| SWFRG024-19 | Fabaceae | *Lotus corniculatus* | UGEAB095-17 | Fabaceae | *Lotus corniculatus* | 90.46 | 503 | FALSE | matK |
| SWFRG024-19 | Fabaceae | *Lotus corniculatus* | GBVX7256-15 | Fabaceae | *Lotus japonicus* | 90.46 | 503 | FALSE | matK |
| SWFRG024-19 | Fabaceae | *Lotus corniculatus* | GBVX3659-15 | Fabaceae | *Lotus filicaulis* | 90.46 | 503 | FALSE | matK |
| SWFRG024-19 | Fabaceae | *Lotus corniculatus* | GBVX3658-15 | Fabaceae | *Lotus burttii* | 90.46 | 503 | FALSE | matK |
| SWFRG024-19 | Fabaceae | *Lotus corniculatus* | GBVX3657-15 | Fabaceae | *Lotus japonicus* | 90.46 | 503 | FALSE | matK |
| SWFRG024-19 | Fabaceae | *Lotus corniculatus* | SDH1158-14 | Fabaceae | *Lotus tenuis* | 90.46 | 503 | FALSE | matK |
| SWFRG024-19 | Fabaceae | *Lotus corniculatus* | SDH1157-14 | Fabaceae | *Lotus corniculatus* | 90.46 | 503 | FALSE | matK |
| SWFRG024-19 | Fabaceae | *Lotus corniculatus* | VPSBC1235-13 | Fabaceae | *Lotus corniculatus* | 90.46 | 503 | FALSE | matK |
| SWFRG024-19 | Fabaceae | *Lotus corniculatus* | VPSBC964-13 | Fabaceae | *Lotus tenuis* | 90.46 | 503 | FALSE | matK |
| SWFRG024-19 | Fabaceae | *Lotus corniculatus* | VPSBC963-13 | Fabaceae | *Lotus krylovii* | 90.46 | 503 | FALSE | matK |
| SWFRG024-19 | Fabaceae | *Lotus corniculatus* | GBVX1797-13 | Fabaceae | *Lotus japonicus* | 90.46 | 503 | FALSE | matK |
| SWFRG024-19 | Fabaceae | *Lotus corniculatus* | GBVR1886-13 | Fabaceae | *Lotus japonicus* | 90.46 | 503 | FALSE | matK |
| SWFRG024-19 | Fabaceae | *Lotus corniculatus* | PLGE037-13 | Fabaceae | *Lotus corniculatus* | 90.46 | 503 | FALSE | matK |
| SWFRG024-19 | Fabaceae | *Lotus corniculatus* | VPSBC379-13 | Fabaceae | *Lotus corniculatus* | 90.46 | 503 | FALSE | matK |
| SWFRG024-19 | Fabaceae | *Lotus corniculatus* | VPSBC269-12 | Fabaceae | *Lotus corniculatus* | 90.46 | 503 | FALSE | matK |
| SWFRG024-19 | Fabaceae | *Lotus corniculatus* | VPSBC243-12 | Fabaceae | *Lotus corniculatus* | 90.46 | 503 | FALSE | matK |
| SWFRG024-19 | Fabaceae | *Lotus corniculatus* | VPSBC208-12 | Fabaceae | *Lotus corniculatus* | 90.46 | 503 | FALSE | matK |
| SWFRG024-19 | Fabaceae | *Lotus corniculatus* | POWNA1011-12 | Fabaceae | *Lotus pedunculatus* | 90.46 | 503 | FALSE | matK |
| SWFRG024-19 | Fabaceae | *Lotus corniculatus* | GBVO1537-11 | Fabaceae | *Lotus corniculatus* | 90.46 | 503 | FALSE | matK |
| SWFRG025-19 | Poaceae | *Lolium perenne* | GBVS364-13 | Poaceae | *Cynosurus cristatus* | 99.52 | 1520 | FALSE | matK |
| SWFRG026-19 | Fabaceae | *Trifolium repens* | GBVO4990-11 | Fabaceae | *Trifolium beckwithii* | 99.05 | 750 | FALSE | matK |
| SWFRG026-19 | Fabaceae | *Trifolium repens* | GBVP076-11 | Fabaceae | *Trifolium thalii* | 99.05 | 750 | FALSE | matK |
| SWFRG026-19 | Fabaceae | *Trifolium repens* | GBVP053-11 | Fabaceae | *Trifolium repens* | 99.05 | 750 | FALSE | matK |
| SWFRG027-19 | Poaceae | *Poa pratensis* | GBVX7900-15 | Poaceae | *Triticum macha* | 99.18 | 878 | FALSE | matK |
| SWFRG027-19 | Poaceae | *Poa pratensis* | GBVX7860-15 | Poaceae | *Triticum turgidum* | 99.18 | 878 | FALSE | matK |
| SWFRG027-19 | Poaceae | *Poa pratensis* | GBVX7728-15 | Poaceae | *Triticum macha* | 99.18 | 878 | FALSE | matK |
| SWFRG027-19 | Poaceae | *Poa pratensis* | GBVX7423-15 | Poaceae | *Triticum aestivum* | 99.18 | 878 | FALSE | matK |
| SWFRG027-19 | Poaceae | *Poa pratensis* | GBVX7248-15 | Poaceae | *Triticum turgidum* | 99.18 | 878 | FALSE | matK |
| SWFRG027-19 | Poaceae | *Poa pratensis* | GBVX6329-15 | Poaceae | *Triticum aestivum* | 99.18 | 878 | FALSE | matK |
| SWFRG027-19 | Poaceae | *Poa pratensis* | GBVX6328-15 | Poaceae | *Triticum turgidum* | 99.18 | 878 | FALSE | matK |
| SWFRG027-19 | Poaceae | *Poa pratensis* | GBVX6327-15 | Poaceae | *Triticum turgidum* | 99.18 | 878 | FALSE | matK |
| SWFRG027-19 | Poaceae | *Poa pratensis* | GBVX6326-15 | Poaceae | *Triticum turgidum* | 99.18 | 878 | FALSE | matK |
| SWFRG027-19 | Poaceae | *Poa pratensis* | GBVX6325-15 | Poaceae | *Triticum turgidum* | 99.18 | 878 | FALSE | matK |
| SWFRG027-19 | Poaceae | *Poa pratensis* | GBVX6324-15 | Poaceae | *Triticum turgidum* | 99.18 | 878 | FALSE | matK |
| SWFRG027-19 | Poaceae | *Poa pratensis* | GBVX6323-15 | Poaceae | *Triticum turgidum* | 99.18 | 878 | FALSE | matK |
| SWFRG027-19 | Poaceae | *Poa pratensis* | GBVX6322-15 | Poaceae | *Triticum aestivum* | 99.18 | 878 | FALSE | matK |
| SWFRG027-19 | Poaceae | *Poa pratensis* | GBVX6295-15 | Poaceae | *Triticum aestivum* | 99.18 | 878 | FALSE | matK |
| SWFRG027-19 | Poaceae | *Poa pratensis* | GBVX1386-13 | Poaceae | *Triticum aestivum* | 99.18 | 878 | FALSE | matK |
| SWFRG027-19 | Poaceae | *Poa pratensis* | MSTH026-12 | Poaceae | *Triticum aestivum* | 99.18 | 878 | FALSE | matK |
| SWFRG027-19 | Poaceae | *Poa pratensis* | GBVD4923-11 | Poaceae | *Triticum turgidum* | 99.18 | 878 | FALSE | matK |
| SWFRG027-19 | Poaceae | *Poa pratensis* | GBVD4922-11 | Poaceae | *Triticum turgidum* | 99.18 | 878 | FALSE | matK |
| SWFRG027-19 | Poaceae | *Poa pratensis* | GBVD4921-11 | Poaceae | *Triticum turgidum* | 99.18 | 878 | FALSE | matK |
| SWFRG027-19 | Poaceae | *Poa pratensis* | GBVD4918-11 | Poaceae | *Triticum turgidum* | 99.18 | 878 | FALSE | matK |
| SWFRG027-19 | Poaceae | *Poa pratensis* | GBVD4917-11 | Poaceae | *Triticum turgidum* | 99.18 | 878 | FALSE | matK |
| SWFRG027-19 | Poaceae | *Poa pratensis* | GBVD4916-11 | Poaceae | *Triticum turgidum* | 99.18 | 878 | FALSE | matK |
| SWFRG027-19 | Poaceae | *Poa pratensis* | GBVD4915-11 | Poaceae | *Triticum turgidum* | 99.18 | 878 | FALSE | matK |
| SWFRG027-19 | Poaceae | *Poa pratensis* | GBVD4903-11 | Poaceae | *Triticum spelta* | 99.18 | 878 | FALSE | matK |
| SWFRG027-19 | Poaceae | *Poa pratensis* | GBVD4880-11 | Poaceae | *Triticum ispahanicum* | 99.18 | 878 | FALSE | matK |
| SWFRG027-19 | Poaceae | *Poa pratensis* | GBVD4866-11 | Poaceae | *Triticum aestivum* | 99.18 | 878 | FALSE | matK |
| SWFRG027-19 | Poaceae | *Poa pratensis* | GRASS1138-07 | Poaceae | *Triticum aestivum* | 99.18 | 878 | FALSE | matK |
| SWFRG027-19 | Poaceae | *Poa pratensis* | KSR158-07 | Poaceae | *Triticum aestivum* | 99.18 | 878 | FALSE | matK |
| SWFRG028-19 | Fabaceae | *Onobrychis viciifolia* | VPSBC974-13 | Fabaceae | *Onobrychis viciifolia* | 93.7 | 841 | FALSE | matK |
| SWFRG028-19 | Fabaceae | *Onobrychis viciifolia* | GBVU2406-13 | Fabaceae | *Onobrychis viciifolia* | 93.7 | 841 | FALSE | matK |
| SWFRG028-19 | Fabaceae | *Onobrychis viciifolia* | GBVU1464-13 | Fabaceae | *Onobrychis argentea* | 93.7 | 841 | FALSE | matK |
| SWFRG028-19 | Fabaceae | *Onobrychis viciifolia* | GBVO2337-11 | Fabaceae | *Onobrychis montana* | 93.7 | 841 | FALSE | matK |
| SWFRG030-19 | Poaceae | *Cynosurus cristatus* | SWFRG046-19 | Poaceae | *Cynosurus cristatus* | 99.81 | 942 | TRUE | matK |
| SWFRG030-19 | Poaceae | *Cynosurus cristatus* | GBVS364-13 | Poaceae | *Cynosurus cristatus* | 99.81 | 942 | TRUE | matK |
| SWFRG030-19 | Poaceae | *Cynosurus cristatus* | GBVD3263-11 | Poaceae | *Cynosurus cristatus* | 99.81 | 942 | TRUE | matK |
| SWFRG030-19 | Poaceae | *Cynosurus cristatus* | GRASS1174-07 | Poaceae | *Cynosurus cristatus* | 99.81 | 942 | TRUE | matK |
| SWFRG030-19 | Poaceae | *Cynosurus cristatus* | GRASS1038-07 | Poaceae | *Cynosurus cristatus* | 99.81 | 942 | TRUE | matK |
| SWFRG031-19 | Poaceae | *Arrhenatherum elatius* | GBVS364-13 | Poaceae | *Cynosurus cristatus* | 99.27 | 1478 | FALSE | matK |
| SWFRG033-19 | Poaceae | *Trisetum flavescens* | SWFRG034-19 | Poaceae | *Trisetum flavescens* | 98.81 | 1048 | TRUE | matK |
| SWFRG034-19 | Poaceae | *Trisetum flavescens* | GBVD4607-11 | Poaceae | *Rostraria pubescens* | 99.66 | 1592 | FALSE | matK |
| SWFRG034-19 | Poaceae | *Trisetum flavescens* | GBVD4606-11 | Poaceae | *Rostraria cristata* | 99.66 | 1592 | FALSE | matK |
| SWFRG035-19 | Fabaceae | *Trifolium pratense* | UGEAB102-17 | Fabaceae | *Trifolium pratense* | 98.92 | 990 | TRUE | matK |
| SWFRG035-19 | Fabaceae | *Trifolium pratense* | VEMSH166-13 | Fabaceae | *Trifolium pratense* | 98.92 | 990 | TRUE | matK |
| SWFRG035-19 | Fabaceae | *Trifolium pratense* | GBVP046-11 | Fabaceae | *Trifolium pratense* | 98.92 | 990 | TRUE | matK |
| SWFRG035-19 | Fabaceae | *Trifolium pratense* | GBVP045-11 | Fabaceae | *Trifolium pratense* | 98.92 | 990 | TRUE | matK |
| SWFRG035-19 | Fabaceae | *Trifolium pratense* | GBVP044-11 | Fabaceae | *Trifolium pratense* | 98.92 | 990 | TRUE | matK |
| SWFRG035-19 | Fabaceae | *Trifolium pratense* | GBVP041-11 | Fabaceae | *Trifolium pratense* | 98.92 | 990 | TRUE | matK |
| SWFRG036-19 | Fabaceae | *Trifolium pratense* | SWFRG035-19 | Fabaceae | *Trifolium pratense* | 99.07 | 769 | TRUE | matK |
| SWFRG037-19 | Fabaceae | *Medicago sativa* | SWFRG038-19 | Fabaceae | *Medicago sativa* | 94.25 | 654 | TRUE | matK |
| SWFRG038-19 | Fabaceae | *Medicago sativa* | GBVU2389-13 | Fabaceae | *Medicago cretacea* | 99.53 | 782 | FALSE | matK |
| SWFRG038-19 | Fabaceae | *Medicago sativa* | GBVR1612-13 | Fabaceae | *Medicago sativa* | 99.53 | 782 | FALSE | matK |
| SWFRG038-19 | Fabaceae | *Medicago sativa* | FOND126-12 | Fabaceae | *Medicago lupulina* | 99.53 | 782 | FALSE | matK |
| SWFRG038-19 | Fabaceae | *Medicago sativa* | GBVO1768-11 | Fabaceae | *Medicago sativa* | 99.53 | 782 | FALSE | matK |
| SWFRG038-19 | Fabaceae | *Medicago sativa* | GBVO1767-11 | Fabaceae | *Medicago sativa* | 99.53 | 782 | FALSE | matK |
| SWFRG038-19 | Fabaceae | *Medicago sativa* | GBVO1756-11 | Fabaceae | *Medicago prostrata* | 99.53 | 782 | FALSE | matK |
| SWFRG038-19 | Fabaceae | *Medicago sativa* | GBVO1748-11 | Fabaceae | *Medicago papillosa* | 99.53 | 782 | FALSE | matK |
| SWFRG038-19 | Fabaceae | *Medicago sativa* | KSR460-08 | Fabaceae | *Medicago sativa* | 99.53 | 782 | FALSE | matK |
| SWFRG038-19 | Fabaceae | *Medicago sativa* | PLNOR093-08 | Fabaceae | *Medicago sativa* | 99.53 | 782 | FALSE | matK |
| SWFRG039-19 | Fabaceae | *Lotus corniculatus* | SWFRG040-19 | Fabaceae | *Lotus corniculatus* | 98.43 | 893 | TRUE | matK |
| SWFRG040-19 | Fabaceae | *Lotus corniculatus* | GBVX3659-15 | Fabaceae | *Lotus filicaulis* | 99.72 | 1286 | FALSE | matK |
| SWFRG041-19 | Fabaceae | *Trifolium repens* | SDH3402-15 | Fabaceae | *Trifolium repens* | 90.44 | 621 | TRUE | matK |
| SWFRG041-19 | Fabaceae | *Trifolium repens* | SERC034-14 | Fabaceae | *Trifolium repens* | 90.44 | 621 | TRUE | matK |
| SWFRG041-19 | Fabaceae | *Trifolium repens* | VPSBC1525-14 | Fabaceae | *Trifolium repens* | 90.44 | 621 | TRUE | matK |
| SWFRG041-19 | Fabaceae | *Trifolium repens* | VPSBC1413-14 | Fabaceae | *Trifolium repens* | 90.44 | 621 | TRUE | matK |
| SWFRG041-19 | Fabaceae | *Trifolium repens* | PIM180-14 | Fabaceae | *Trifolium repens* | 90.44 | 621 | TRUE | matK |
| SWFRG041-19 | Fabaceae | *Trifolium repens* | VPSBC995-13 | Fabaceae | *Trifolium repens* | 90.44 | 621 | TRUE | matK |
| SWFRG041-19 | Fabaceae | *Trifolium repens* | GBVS1253-13 | Fabaceae | *Trifolium repens* | 90.44 | 621 | TRUE | matK |
| SWFRG041-19 | Fabaceae | *Trifolium repens* | PLGE039-13 | Fabaceae | *Trifolium repens* | 90.44 | 621 | TRUE | matK |
| SWFRG041-19 | Fabaceae | *Trifolium repens* | GBVP056-11 | Fabaceae | *Trifolium repens* | 90.44 | 621 | TRUE | matK |
| SWFRG041-19 | Fabaceae | *Trifolium repens* | GBVP055-11 | Fabaceae | *Trifolium repens* | 90.44 | 621 | TRUE | matK |
| SWFRG041-19 | Fabaceae | *Trifolium repens* | GBVP053-11 | Fabaceae | *Trifolium repens* | 90.44 | 621 | TRUE | matK |
| SWFRG041-19 | Fabaceae | *Trifolium repens* | GBVP048-11 | Fabaceae | *Trifolium repens* | 90.44 | 621 | TRUE | matK |
| SWFRG042-19 | Fabaceae | *Trifolium repens* | GBVO4990-11 | Fabaceae | *Trifolium beckwithii* | 98.2 | 773 | FALSE | matK |
| SWFRG042-19 | Fabaceae | *Trifolium repens* | GBVP076-11 | Fabaceae | *Trifolium thalii* | 98.2 | 773 | FALSE | matK |
| SWFRG042-19 | Fabaceae | *Trifolium repens* | GBVP053-11 | Fabaceae | *Trifolium repens* | 98.2 | 773 | FALSE | matK |
| SWFRG043-19 | Fabaceae | *Onobrychis viciifolia* | GBVU2406-13 | Fabaceae | *Onobrychis viciifolia* | 95.18 | 959 | TRUE | matK |
| SWFRG044-19 | Fabaceae | *Onobrychis viciifolia* | GBVU2406-13 | Fabaceae | *Onobrychis viciifolia* | 98.13 | 1229 | TRUE | matK |
| SWFRG045-19 | Poaceae | *Cynosurus cristatus* | GBVS364-13 | Poaceae | *Cynosurus cristatus* | 99.25 | 957 | TRUE | matK |
| SWFRG045-19 | Poaceae | *Cynosurus cristatus* | GBVD3263-11 | Poaceae | *Cynosurus cristatus* | 99.25 | 957 | TRUE | matK |
| SWFRG045-19 | Poaceae | *Cynosurus cristatus* | GRASS1174-07 | Poaceae | *Cynosurus cristatus* | 99.25 | 957 | TRUE | matK |
| SWFRG045-19 | Poaceae | *Cynosurus cristatus* | GRASS1038-07 | Poaceae | *Cynosurus cristatus* | 99.25 | 957 | TRUE | matK |
| SWFRG046-19 | Poaceae | *Cynosurus cristatus* | GBVS364-13 | Poaceae | *Cynosurus cristatus* | 99.54 | 1585 | TRUE | matK |
| SWFRG047-19 | Poaceae | *Phleum pratense* | VPL104-13 | Poaceae | *Phleum pratense* | 99.61 | 939 | TRUE | matK |
| SWFRG047-19 | Poaceae | *Phleum pratense* | GBVD4419-11 | Poaceae | *Phleum pratense* | 99.61 | 939 | TRUE | matK |
| SWFRG047-19 | Poaceae | *Phleum pratense* | GBVD4416-11 | Poaceae | *Phleum pratense* | 99.61 | 939 | TRUE | matK |
| SWFRG048-19 | Poaceae | *Phleum pratense* | GBVS364-13 | Poaceae | *Cynosurus cristatus* | 99.81 | 950 | FALSE | matK |
| SWFRG001-19 | Poaceae | *Dactylis glomerata* | SERC022-14 | Poaceae | *Dactylis glomerata* | 98.48 | 918 | TRUE | trnH-psbA |
| SWFRG001-19 | Poaceae | *Dactylis glomerata* | SCBI233-14 | Poaceae | *Dactylis glomerata* | 98.48 | 918 | TRUE | trnH-psbA |
| SWFRG001-19 | Poaceae | *Dactylis glomerata* | GRASS953-07 | Poaceae | *Dactylis glomerata* | 98.48 | 918 | TRUE | trnH-psbA |
| SWFRG001-19 | Poaceae | *Dactylis glomerata* | KSR057-07 | Poaceae | *Dactylis glomerata* | 98.48 | 918 | TRUE | trnH-psbA |
| SWFRG002-19 | Poaceae | *Dactylis glomerata* | SCBI193-14 | Poaceae | *Dactylis glomerata* | 99.65 | 1055 | TRUE | trnH-psbA |
| SWFRG003-19 | Poaceae | *Festuca pratensis* | KSR314-07 | Poaceae | *Festuca arundinacea* | 99.64 | 1013 | FALSE | trnH-psbA |
| SWFRG004-19 | Poaceae | *Festuca pratensis* | KSR314-07 | Poaceae | *Festuca arundinacea* | 99.12 | 1022 | FALSE | trnH-psbA |
| SWFRG005-19 | Poaceae | *Lolium multiflorum* | SWFRG021-19 | Poaceae | *Lolium multiflorum* | 99.44 | 972 | TRUE | trnH-psbA |
| SWFRG006-19 | Poaceae | *Lolium multiflorum* | KSR314-07 | Poaceae | *Festuca arundinacea* | 97.55 | 987 | FALSE | trnH-psbA |
| SWFRG007-19 | Poaceae | *Festuca rubra* | GRASS1057-07 | Poaceae | *Festuca rubra* | 88.13 | 364 | FALSE | trnH-psbA |
| SWFRG007-19 | Poaceae | *Festuca rubra* | GRASS609-07 | Poaceae | *Festuca brachyphylla* | 88.13 | 364 | FALSE | trnH-psbA |
| SWFRG007-19 | Poaceae | *Festuca rubra* | GRASS578-07 | Poaceae | *Festuca saximontana* | 88.13 | 364 | FALSE | trnH-psbA |
| SWFRG007-19 | Poaceae | *Festuca rubra* | GRASS265-07 | Poaceae | *Festuca saximontana* | 88.13 | 364 | FALSE | trnH-psbA |
| SWFRG007-19 | Poaceae | *Festuca rubra* | GRASS244-07 | Poaceae | *Festuca rubra* | 88.13 | 364 | FALSE | trnH-psbA |
| SWFRG007-19 | Poaceae | *Festuca rubra* | GRASS175-07 | Poaceae | *Festuca rubra* | 88.13 | 364 | FALSE | trnH-psbA |
| SWFRG007-19 | Poaceae | *Festuca rubra* | GRASS156-07 | Poaceae | *Festuca rubra* | 88.13 | 364 | FALSE | trnH-psbA |
| SWFRG007-19 | Poaceae | *Festuca rubra* | GRASS130-07 | Poaceae | *Festuca saximontana* | 88.13 | 364 | FALSE | trnH-psbA |
| SWFRG008-19 | Poaceae | *Festuca rubra* | GRASS316-07 | Poaceae | *Festuca rubra* | 98.95 | 1018 | TRUE | trnH-psbA |
| SWFRG009-19 | Poaceae | *Lolium perenne* | GRASS1020-07 | Poaceae | *Lolium perenne* | 98.15 | 952 | TRUE | trnH-psbA |
| SWFRG009-19 | Poaceae | *Lolium perenne* | KSR116-07 | Poaceae | *Lolium perenne* | 98.15 | 952 | TRUE | trnH-psbA |
| SWFRG009-19 | Poaceae | *Lolium perenne* | GRASS808-07 | Poaceae | *Lolium perenne* | 98.15 | 952 | TRUE | trnH-psbA |
| SWFRG010-19 | Poaceae | *Lolium perenne* | GRASS1020-07 | Poaceae | *Lolium perenne* | 95.8 | 942 | TRUE | trnH-psbA |
| SWFRG010-19 | Poaceae | *Lolium perenne* | GRASS808-07 | Poaceae | *Lolium perenne* | 95.8 | 942 | TRUE | trnH-psbA |
| SWFRG011-19 | Poaceae | *Poa pratensis* | GRASS1217-07 | Poaceae | *Poa pratensis* | 98.71 | 957 | FALSE | trnH-psbA |
| SWFRG011-19 | Poaceae | *Poa pratensis* | KSR067-07 | Poaceae | *Poa pratensis* | 98.71 | 957 | FALSE | trnH-psbA |
| SWFRG011-19 | Poaceae | *Poa pratensis* | GRASS561-07 | Poaceae | *Poa pratensis* | 98.71 | 957 | FALSE | trnH-psbA |
| SWFRG011-19 | Poaceae | *Poa pratensis* | GRASS400-07 | Poaceae | *Poa arctica* | 98.71 | 957 | FALSE | trnH-psbA |
| SWFRG011-19 | Poaceae | *Poa pratensis* | GRASS341-07 | Poaceae | *Poa arctica* | 98.71 | 957 | FALSE | trnH-psbA |
| SWFRG011-19 | Poaceae | *Poa pratensis* | GRASS333-07 | Poaceae | *Poa arctica* | 98.71 | 957 | FALSE | trnH-psbA |
| SWFRG012-19 | Poaceae | *Poa pratensis* | GRASS1217-07 | Poaceae | *Poa pratensis* | 99.13 | 1031 | FALSE | trnH-psbA |
| SWFRG012-19 | Poaceae | *Poa pratensis* | KSR067-07 | Poaceae | *Poa pratensis* | 99.13 | 1031 | FALSE | trnH-psbA |
| SWFRG012-19 | Poaceae | *Poa pratensis* | GRASS561-07 | Poaceae | *Poa pratensis* | 99.13 | 1031 | FALSE | trnH-psbA |
| SWFRG012-19 | Poaceae | *Poa pratensis* | GRASS400-07 | Poaceae | *Poa arctica* | 99.13 | 1031 | FALSE | trnH-psbA |
| SWFRG012-19 | Poaceae | *Poa pratensis* | GRASS341-07 | Poaceae | *Poa arctica* | 99.13 | 1031 | FALSE | trnH-psbA |
| SWFRG012-19 | Poaceae | *Poa pratensis* | GRASS333-07 | Poaceae | *Poa arctica* | 99.13 | 1031 | FALSE | trnH-psbA |
| SWFRG015-19 | Poaceae | *Arrhenatherum elatius* | GRASS1024-07 | Poaceae | *Arrhenatherum elatius* | 95.74 | 780 | TRUE | trnH-psbA |
| SWFRG017-19 | Poaceae | *Dactylis glomerata* | SERC022-14 | Poaceae | *Dactylis glomerata* | 99.64 | 1027 | TRUE | trnH-psbA |
| SWFRG017-19 | Poaceae | *Dactylis glomerata* | SCBI233-14 | Poaceae | *Dactylis glomerata* | 99.64 | 1027 | TRUE | trnH-psbA |
| SWFRG017-19 | Poaceae | *Dactylis glomerata* | GRASS953-07 | Poaceae | *Dactylis glomerata* | 99.64 | 1027 | TRUE | trnH-psbA |
| SWFRG017-19 | Poaceae | *Dactylis glomerata* | KSR057-07 | Poaceae | *Dactylis glomerata* | 99.64 | 1027 | TRUE | trnH-psbA |
| SWFRG018-19 | Poaceae | *Trisetum flavescens* | SWFRG033-19 | Poaceae | *Trisetum flavescens* | 99.65 | 1042 | TRUE | trnH-psbA |
| SWFRG019-19 | Poaceae | *Festuca pratensis* | SWFRG004-19 | Poaceae | *Festuca pratensis* | 98.02 | 957 | TRUE | trnH-psbA |
| SWFRG020-19 | Fabaceae | *Trifolium pratense* | SWFRG036-19 | Fabaceae | *Trifolium pratense* | 99.27 | 741 | TRUE | trnH-psbA |
| SWFRG020-19 | Fabaceae | *Trifolium pratense* | SWFRG035-19 | Fabaceae | *Trifolium pratense* | 99.27 | 741 | TRUE | trnH-psbA |
| SWFRG020-19 | Fabaceae | *Trifolium pratense* | SERC046-14 | Fabaceae | *Trifolium pratense* | 99.27 | 741 | TRUE | trnH-psbA |
| SWFRG021-19 | Poaceae | *Lolium multiflorum* | SWFRG004-19 | Poaceae | *Festuca pratensis* | 99.64 | 1003 | FALSE | trnH-psbA |
| SWFRG022-19 | Fabaceae | *Medicago sativa* | SWFRG037-19 | Fabaceae | *Medicago sativa* | 87.55 | 311 | TRUE | trnH-psbA |
| SWFRG023-19 | Poaceae | *Festuca rubra* | GRASS316-07 | Poaceae | *Festuca rubra* | 97.97 | 1020 | TRUE | trnH-psbA |
| SWFRG024-19 | Fabaceae | *Lotus corniculatus* | SWFRG040-19 | Fabaceae | *Lotus corniculatus* | 97.65 | 507 | TRUE | trnH-psbA |
| SWFRG024-19 | Fabaceae | *Lotus corniculatus* | WABLK784-13 | Fabaceae | *Lotus corniculatus* | 97.65 | 507 | TRUE | trnH-psbA |
| SWFRG024-19 | Fabaceae | *Lotus corniculatus* | WABLK775-13 | Fabaceae | *Lotus corniculatus* | 97.65 | 507 | TRUE | trnH-psbA |
| SWFRG024-19 | Fabaceae | *Lotus corniculatus* | KSR068-07 | Fabaceae | *Lotus corniculatus* | 97.65 | 507 | TRUE | trnH-psbA |
| SWFRG025-19 | Poaceae | *Lolium perenne* | GRASS953-07 | Poaceae | *Dactylis glomerata* | 97.61 | 641 | FALSE | trnH-psbA |
| SWFRG026-19 | Fabaceae | *Trifolium repens* | SWFRG041-19 | Fabaceae | *Trifolium repens* | 99.33 | 813 | TRUE | trnH-psbA |
| SWFRG028-19 | Fabaceae | *Onobrychis viciifolia* | SWFRG044-19 | Fabaceae | *Onobrychis viciifolia* | 99.29 | 508 | TRUE | trnH-psbA |
| SWFRG028-19 | Fabaceae | *Onobrychis viciifolia* | SWFRG043-19 | Fabaceae | *Onobrychis viciifolia* | 99.29 | 508 | TRUE | trnH-psbA |
| SWFRG029-19 | Poaceae | *Alopecurus pratensis* | GRASS353-07 | Poaceae | *Arctophila fulva* | 99.12 | 1024 | FALSE | trnH-psbA |
| SWFRG030-19 | Poaceae | *Cynosurus cristatus* | SWFRG046-19 | Poaceae | *Cynosurus cristatus* | 98.92 | 826 | TRUE | trnH-psbA |
| SWFRG033-19 | Poaceae | *Trisetum flavescens* | SWFRG018-19 | Poaceae | *Trisetum flavescens* | 99.65 | 1042 | TRUE | trnH-psbA |
| SWFRG034-19 | Poaceae | *Trisetum flavescens* | SWFRG033-19 | Poaceae | *Trisetum flavescens* | 99.82 | 1037 | TRUE | trnH-psbA |
| SWFRG035-19 | Fabaceae | *Trifolium pratense* | SWFRG036-19 | Fabaceae | *Trifolium pratense* | 99.59 | 885 | TRUE | trnH-psbA |
| SWFRG036-19 | Fabaceae | *Trifolium pratense* | SWFRG035-19 | Fabaceae | *Trifolium pratense* | 99.59 | 885 | TRUE | trnH-psbA |
| SWFRG037-19 | Fabaceae | *Medicago sativa* | SWFRG038-19 | Fabaceae | *Medicago sativa* | 97.96 | 765 | TRUE | trnH-psbA |
| SWFRG038-19 | Fabaceae | *Medicago sativa* | SWFRG037-19 | Fabaceae | *Medicago sativa* | 97.96 | 765 | TRUE | trnH-psbA |
| SWFRG039-19 | Fabaceae | *Lotus corniculatus* | SWFRG040-19 | Fabaceae | *Lotus corniculatus* | 99.27 | 745 | TRUE | trnH-psbA |
| SWFRG040-19 | Fabaceae | *Lotus corniculatus* | SWFRG039-19 | Fabaceae | *Lotus corniculatus* | 99.27 | 745 | TRUE | trnH-psbA |
| SWFRG041-19 | Fabaceae | *Trifolium repens* | VPL086-13 | Fabaceae | *Trifolium repens* | 99.56 | 828 | TRUE | trnH-psbA |
| SWFRG042-19 | Fabaceae | *Trifolium repens* | VPL086-13 | Fabaceae | *Trifolium repens* | 99.78 | 821 | TRUE | trnH-psbA |
| SWFRG043-19 | Fabaceae | *Onobrychis viciifolia* | SWFRG044-19 | Fabaceae | *Onobrychis viciifolia* | 100 | 523 | TRUE | trnH-psbA |
| SWFRG044-19 | Fabaceae | *Onobrychis viciifolia* | SWFRG043-19 | Fabaceae | *Onobrychis viciifolia* | 100 | 523 | TRUE | trnH-psbA |
| SWFRG045-19 | Poaceae | *Cynosurus cristatus* | SWFRG046-19 | Poaceae | *Cynosurus cristatus* | 100 | 1042 | TRUE | trnH-psbA |
| SWFRG046-19 | Poaceae | *Cynosurus cristatus* | SWFRG045-19 | Poaceae | *Cynosurus cristatus* | 100 | 1042 | TRUE | trnH-psbA |
| SWFRG047-19 | Poaceae | *Phleum pratense* | VPL104-13 | Poaceae | *Phleum pratense* | 99.83 | 1072 | TRUE | trnH-psbA |
| SWFRG047-19 | Poaceae | *Phleum pratense* | GRASS1089-07 | Poaceae | *Phleum pratense* | 99.83 | 1072 | TRUE | trnH-psbA |
| SWFRG047-19 | Poaceae | *Phleum pratense* | KSR091-07 | Poaceae | *Phleum pratense* | 99.83 | 1072 | TRUE | trnH-psbA |

**References**

1. Soltis PS, Soltis DE, Smiley CJ. An rbcL sequence from a Miocene Taxodium (bald cypress). Proc Natl Acad Sci U S A. 1992;89:449–51. http://www.ncbi.nlm.nih.gov/pubmed/1729716%0Ahttp://www.pubmedcentral.nih.gov/articlerender.fcgi?artid=PMC48255.

2. Levin RA, Wagner WL, Hoch PC, Nepokroeff M, Pires JC, Zimmer EA, et al. Family-level relationships of Onagraceae based on chloroplast rbc L and ndh F data. Am J Bot. 2003;90:107–15. doi:10.3732/ajb.90.1.107.

3. Kress WJ, Erickson DL. A Two-Locus Global DNA Barcode for Land Plants: The Coding rbcL Gene Complements the Non-Coding trnH-psbA Spacer Region. PLoS One. 2007;2:e508. doi:10.1371/journal.pone.0000508.

4. Kress WJ, Erickson DL, Jones FA, Swenson NG, Perez R, Sanjur O, et al. Plant DNA barcodes and a community phylogeny of a tropical forest dynamics plot in Panama. Proc Natl Acad Sci U S A. 2009;106:18621–6. doi:10.1073/pnas.0909820106.

5. Fofana B, Harvengt L, Baudoin JP, Du Jardin P. New primers for the polymerase chain amplification of cpDNA intergenic spacers in Phaseolus phylogeny. Belgian J Bot. 1997;129:118–22.

6. Dunning LT, Savolainen V. Broad-scale amplification of matK for DNA barcoding plants, a technical note. Bot J Linn Soc. 2010;164:1–9. doi:10.1111/j.1095-8339.2010.01071.x.

7. Wang X-R, Tsumura Y, Yoshimaru H, Nagasaka K, Szmidt AE. Phylogenetic Relationships of Eurasian Pines (Pinus, Pinaceae) Based on Chloroplast rbcL, matK, rpl20-rps18 Spacer, and trnV Intron Sequences. Am J Bot. 1999;86:1742. doi:10.2307/2656672.

8. Sang T, Crawford DJ, Stuessy TF. Chloroplast DNA phylogeny, reticulate evolution, and biogeography of Paeonia (Paeoniaceae). Am J Bot. 1997;84:1120–36. doi:10.2307/2446155.

9. Simpson B, Tate JA. Paraphyly of Tarasa (Malvaceae) and diverse origins of the polyploid species. Syst Bot. 2003;28:723–37. http://www.bioone.org/doi/abs/10.1043/02-64.1.

10. Ratnasingham S, Hebert PDN. BARCODING: bold: The Barcode of Life Data System (http://www.barcodinglife.org). Mol Ecol Notes. 2007;7:355–64. doi:10.1111/j.1471-8286.2007.01678.x.
